# Research Progress on Micro/Nanopore Flow Behavior

**DOI:** 10.3390/molecules30081807

**Published:** 2025-04-17

**Authors:** Jinbo Yu, Meng Du, Yapu Zhang, Xinliang Chen, Zhengming Yang

**Affiliations:** 1 University of Chinese Academy of Sciences, Beijing 100049, China; yujinbo23@mails.ucas.ac.cn (J.Y.); chenxinliang20@mails.ucas.ac.cn (X.C.); 2 Institute of Porous Flow and Fluid Mechanics, Chinese Academy of Sciences, Langfang 065007, China; zhangyapu69@petrochina.com.cn; 3 Research Institute of Petroleum Exploration & Development, PetroChina Company Limited, Beijing 100083, China

**Keywords:** microseepage, confined flow behavior, nanoporous media, experimental research, molecular dynamics simulation, lattice Boltzmann method, artificial intelligence, pore-scale modeling

## Abstract

Fluid flow in microporous and nanoporous media exhibits unique behaviors that deviate from classical continuum predictions due to dominant surface forces at small scales. Understanding these microscale flow mechanisms is critical for optimizing unconventional reservoir recovery and other energy applications. This review provides a comparative analysis of the existing literature, highlighting key advances in experimental techniques, theoretical models, and numerical simulations. We discuss how innovative micro/nanofluidic devices and high-resolution imaging methods now enable direct observation of confined flow phenomena, such as slip flow, phase transitions, and non-Darcy behavior. Recent theoretical models have clarified scale-dependent flow regimes by distinguishing microscale effects from macroscopic Darcy flow. Likewise, advanced numerical simulations—including molecular dynamics (MD), lattice Boltzmann methods (LBM), and hybrid multiscale frameworks—capture complex fluid–solid interactions and multiphase dynamics under realistic pressure and wettability conditions. Moreover, the integration of artificial intelligence (e.g., data-driven modeling and physics-informed neural networks) is accelerating data interpretation and multiscale modeling, offering improved predictive capabilities. Through this critical review, key phenomena, such as adsorption layers, fluid–solid interactions, and pore surface heterogeneity, are examined across studies, and persistent challenges are identified. Despite notable progress, challenges remain in replicating true reservoir conditions, bridging microscale and continuum models, and fully characterizing multiphase interface dynamics. By consolidating recent progress and perspectives, this review not only summarizes the state-of-the-art but underscores remaining knowledge gaps and future directions in micro/nanopore flow research.

## 1. Introduction

**Figure 1:**
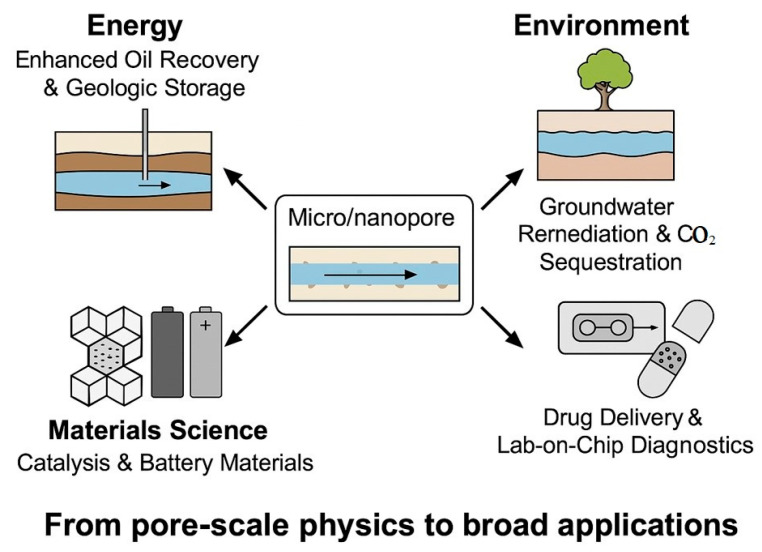
Schematic illustration of typical cross-domain applications enabled by micro/nanopore fluid dynamics.

Research on microscopic seepage plays a crucial role across various energy fields, significantly influencing resource development, utilization, and optimization. For CO_2_ geological storage and enhanced oil recovery, the microscopic seepage mechanism governs the capillary storage, dissolution, diffusion, seepage, and mineral interactions of CO_2_, thereby influencing storage stability, long-term safety, optimizing storage efficiency, and mitigating leakage risks [1]. During geothermal energy extraction, the transport of hot fluids at the microscale is affected by the dual-medium structure of the fracture matrix. Studying its permeability characteristics can optimize the thermal recovery rate, improve the heat transfer efficiency, and extend the life of geothermal reservoirs [2]. Regarding hydrogen energy storage, hydrogen permeation in porous media is governed by capillary action, wettability, and dissolution characteristics. Microscopic permeation research can improve storage recovery, optimize reservoir design, and reduce hydrogen loss [3]. In the field of microscale fuel cells, the micropore structure and permeation characteristics of the catalytic layer determine the transport efficiency and cell performance of reactants (oxygen, hydrogen). Optimizing the micro transport process can improve energy conversion efficiency and enhance the durability of fuel cells [4]. Thus, research on microscopic seepage serves as the foundation for understanding fluid behavior in energy reservoirs and engineering systems. It is also crucial for enhancing energy development efficiency, optimizing reservoir utilization, and improving the stability of emerging energy systems. Existing reviews mostly focus on specific aspects, such as experimental techniques [5], simulation methods [6], nanoscale flow in shale systems [7], or microfluidic applications in chemical EOR (Enhanced Oil Recovery) [8]; however, a comprehensive integration of mechanisms and methodologies across multiple scales has been lacking. By contrast, recent progress in experimental techniques (e.g., high-resolution dynamic imaging), numerical simulations (e.g., grayscale LBM, coupled LBM–MD), and AI-enhanced modeling has been integrated in the present study. These developments, particularly those made in the past three to five years, have addressed the growing demand for more accurate and scalable models in unconventional reservoir characterization, energy storage, and environmental systems, and have thereby provided timely insights for the research community. As illustrated in Figure 1, micro/nanopore fluid dynamics find wide-ranging applications across diverse fields, such as energy, environmental science, life sciences, and materials science.

**Figure 2:**
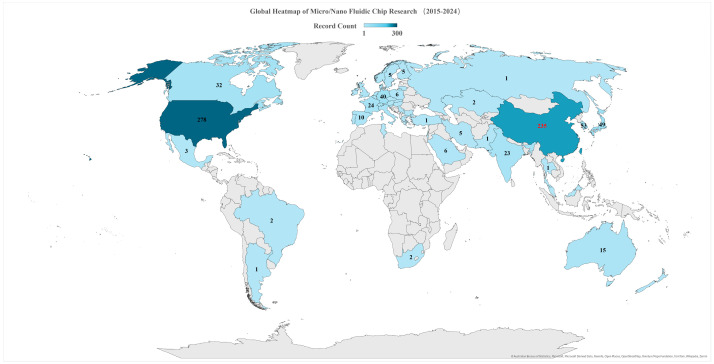
World map showing the number of publications in micro/nanofluidic chip research by country (2015–2024). China is highlighted in red, reflecting its status as the second-largest contributor globally (after the United States) based on a meta-analysis of publication records.

The literature for this review was primarily gathered through structured searches of the Scopus database using specific keywords (e.g., “microfluidic flow”, “nanoscale seepage”, “pore-scale transport”, “molecular simulation in porous media”). In addition to the most recent publications, several earlier yet representative studies were included to provide historical context and trace the evolution of key methodologies. Relevant papers were also identified by cross-referencing recent review articles and high-impact studies, ensuring a thorough survey of the field. Collectively, these efforts yielded a comprehensive collection of pertinent studies and provided a foundation for examining the field’s global research landscape. As shown in Figure 2, a meta-analysis of global publication records from 2015U+2012024 illustrates widespread international activity in micro- and nanofluidic chip research. The United States leads in publication output, and China (highlighted in red) ranks second, underscoring China’s significant role in advancing this field.

### 1.1. Background

In this paper, we use the term “microflows” to refer to fluid flow at the micro- to nanoscale in porous media, while “microfluidic systems” denote lab-scale devices designed to study such flow behaviors.

In recent years, the focus of oil exploration and development has increasingly shifted toward unconventional resources, including shale gas, tight gas, and coalbed methane. The precise characterization of nanoscale pore structures and fluid behavior in minerals has emerged as a critical technique for reservoir evaluation. Fluids in micro/nanoporous media refer to fluids that flow at the microscale (typically ranging from nanometers to micrometers) and exhibit properties such as viscosity, surface tension, wettability, capillary forces, and diffusion effects. These properties often differ from macroscopic fluid mechanics behavior in microenvironments, where surface effects predominantly govern flow rather than inertial forces. Microscale seepage describes the movement of fluids through pores or fractures within structures at scales ranging from nanometers to micrometers. Such seepage is primarily governed by microscale flow properties rather than the conventional seepage laws defined by Darcy’s law. Research on microscopic seepage primarily investigates fluid migration in complex pore networks, oil–water interactions, and the impact of heterogeneous distributions on fluid transport [9]. Microscopic seepage is crucial in various fields of natural science and engineering, including shale gas reservoirs, where migration models are influenced by microscale and fracture mechanisms [10], pore blockage and clearing in porous media during groundwater movement [11], and microscale personalized drug delivery [12,13]. Since macroscopic parameters, such as capillary pressure, relative permeability, and inlet pressure, can be quantified through microscopic flow effects, multi-scale seepage processes exhibit greater complexity compared to single-scale fluid flows [14,15,16,17,18]. The intricate pore-throat architecture and multiphase distribution introduce significant uncertainties that affect flow behavior in complex porous media [19]. In the field of tight oil, porous media exhibit a multi-mineral composition and multi-scale characteristics, ranging from nanometers to meters [14,20]. This results in intricate multiphase flow behavior involving various fluid phases (oil–water, oil–gas, gas–water, water–water, oil–CO_2_, oil–CO_2_–water, etc.) within porous media. Understanding the microscale flow patterns in complex porous media is crucial, as it facilitates the study of carbon dioxide mixture pressure while also contributing to theoretical advancements and engineering applications.

### 1.2. Research Status and Problems

Research on microscale flow properties in porous media primarily involves theoretical analysis, experimental investigations, and numerical simulations. Extensive theoretical studies on microfluidics have been conducted prior to experimental investigations. In microscale flow channels, viscous forces predominate over inertial forces. The flow rate is typically determined using the Navier–Stokes equation (N-S equation) based on the Reynolds number. When the wall velocity is zero, the equation simplifies to the Hagen–Poiseuille (HP) equation for analytical solutions [21,22]. Assuming a no-slip condition, Navier proposed that the slip velocity at the wall is proportional to the fluid shear rate [23,24]. At the micro/nano scale, both negative and positive slip phenomena may arise [25,26,27,28,29]. Due to molecular viscous forces, a stable boundary layer forms near the wall, and flow enhancement effects in nanochannels have also been observed [30], or some scholars have also found flow enhancement phenomena in nanochannels [31,32,33,34].

To enhance the understanding of microflow properties, further validation through experimental or numerical simulations is required. Core-scale fluid transport experiments, which integrate online nuclear magnetic resonance coupling and CT scanning, are widely used to study microscale seepage by obtaining T1 and T2 spectra within the core to simultaneously monitor fluid distribution [35,36]. Core-scale experiments can effectively capture the dynamic evolution of microstructure, fluid flow, and distribution within the core. However, these methods rely on the core as the research subject, where pores and throats of various sizes coexist. The resulting fluid transport laws represent averaged results from multiple pore sizes and do not accurately reflect the influence of micro–nano confined spaces on flow. Consequently, they fail to capture the microscopic mechanisms that govern macroscopic flow characteristics. Pore-scale microfluidic experiments address this limitation by visualizing interface dynamics and multiphase distribution, thereby elucidating microscopic fluid transport mechanisms [37]. However, microfluidic chips are limited by factors such as aperture size and experimental conditions, including difficulties in achieving nanoscale microfluidic chips and conducting high-temperature, high-pressure experiments. Additionally, due to manufacturing constraints, the channels within the chips are typically rectangular. Due to observation limitations, nanofluidic chips typically achieve nanoscale dimensions only in the depth direction, while the width remains in the micrometer range. Accurately representing the multiscale flow mechanism of microfluidics beyond three dimensions is challenging [38,39,40]. Furthermore, microfluidic experiments involving nanoscale porous media are costly. Compared to traditional laboratory techniques, microfluidic technology offers several advantages, including low sample consumption, rapid analysis, high automation, elevated heat and mass transfer rates, easy integration, and enhanced safety [41,42]. Currently, two types of microchannels are used for studying micro-percolation: micro/nanotubes and microfluidic chips. Micro/nano round tubes can achieve nanometer-scale confinement in two dimensions, closely resembling the actual rock micropore-throat structure. Commonly used types include quartz capillary [43], nanoscale carbon nanotubes [44], and alumina films [45,46]. Their size is controllable, with the minimum size reaching several nanometers. The channel structure is uniform, and wall wettability is adjustable. Microfluidic chips can be designed with various structures, including T-shaped, cross-shaped, serpentine, and other regular designs, as well as packed beds, multiscale flow networks, and heterogeneous structures, depending on the experimental requirements [38,39,40]. Wetting properties can also be adjusted based on mineral compositions [47,48,49]. However, due to manufacturing constraints, the channels within the chips are typically rectangular. The combination of micro/nanotubes and microfluidic chips provides an effective platform for studying microscopic seepage properties in petroleum reservoirs.

Numerical simulation methods for fluid transport at the micro/nano scale vary depending on the research scale and include the lattice Boltzmann method (LBM), molecular dynamics (MD), and dissipative particle dynamics (DPD), among others. The fundamental principle of the LBM is to provide a bridge between microscale and macroscale transport by treating all particles as a collective system. Their overall motion characteristics are described using distribution functions, while the spatial domain is represented by a grid-based arrangement. Based on the precision level of pore-throat characterization, LBM is classified into pore-scale LBM and representative elementary volume (REV)-scale LBM. The LBM efficiently handles solid–wall interactions; however, additional numerical techniques are required for treating interfacial boundaries between fluids. The LBM is particularly well-suited for applications involving microfluidic chips, porous media, multiphase flow, and nanofluids [50,51]. The core principle of the MD method involves integrating time-dependent functions based on classical mechanical laws to accurately compute the velocity and position of each molecule and atom within the system. MD simulations achieve high accuracy in systems containing a small number of molecules and atoms. MD simulations are particularly useful for investigating nanoscale fluid properties, including viscosity effects, interfacial tension, and wettability. Additionally, MD can simulate multiphase flow, oil–water and solid–liquid interfaces, and surface adsorption phenomena. MD is also applied in studies of surface wettability, nanolubrication, microdroplet transport, and fluid transport characteristics. Furthermore, MD is applicable to high-temperature and high-pressure research. Overall, MD is more effective for analyzing fluid flow in carbon nanotubes; however, its application to microfluidic chips is constrained by excessive computational complexity [52,53]. In general, the selection of research methods depends on experimental objectives, environmental conditions, and other constraints [54,55]. By leveraging their respective advantages, MD–LBM coupling is applied to microfluidic chip research and pore-scale permeation studies [56,57]. The MD–DPD–LBM coupling approach (nano + meso + micro) is employed for cross-scale fluid dynamics, including shale oil and gas transport [58]. Furthermore, researchers often integrate widely used commercial CFD packages with these open-source techniques to exploit their complementary benefits. For instance, ANSYS Fluent (a commercial finite-volume solver, https://www.ansys.com/products/fluids/ansys-fluent, accessed on 4 March 2025) or COMSOL Multiphysics (a finite-element-based platform, https://www.comsol.com/comsol-multiphysics, accessed on 4 March 2025) can be combined with LBM and MD simulations. This hybrid approach combines the stability and extensive validation of commercial solvers with the high-resolution, physics-rich modeling of open-source methods, thereby improving the accuracy and efficiency of micro/nanopore flow simulations.

## 2. Basic Theory of Fluid Properties in Micro/Nanopores

At the microscale, fluid flow behavior is governed by distinct physical mechanisms that significantly deviate from macroscopic flow. Traditional fluid mechanics theories may become inadequate at the microscale due to the effects of surface tension, capillary phenomena, and fluid–solid interactions. Thus, a reassessment of the fundamental principles governing seepage is required. Microfluidic systems typically display laminar flow characteristics at low Reynolds numbers, where interface effects dominate over inertial forces. Moreover, as the scale decreases, slip boundary effects, Knudsen effects, and discontinuities in the medium become more pronounced, necessitating the incorporation of advanced numerical simulation techniques, including molecular dynamics and the lattice Boltzmann method (LBM), for an accurate depiction of the fluid transport behavior at the micro/nano scale in microscale seepage studies.

### 2.1. Difference Between Microflow and Macro Flow

The distinction between microfluidics and macroscopic fluid flow can no longer be solely determined by Reynolds numbers. Compared to the properties derived from traditional fluid mechanics theories and macroscopic flow models, microfluidic behavior exhibits substantial differences in microscale flow properties in scale effects, flow regimes, inertial and viscous forces, interface interactions, and thermodynamic effects, all of which are influenced by surface tension, capillary phenomena, and fluid–solid interactions [59,60].

### 2.2. Characteristics of Microfluidic Systems

Microfluidic systems generally operate at extremely low Reynolds numbers (*Re*), leading to laminar flow instead of turbulence.

(1) $$Re=\frac{\rho uL}{\mu }$$

where *ρ* is the fluid density, *u* is the flow velocity, *L* is the characteristic length (such as pipeline diameter), and *μ* is the fluid dynamic viscosity [61]. At the microscale (*Re* ≤ 1), laminar flow predominates. Therefore, in microfluidic chips, fluids predominantly exhibit laminar flow, enabling precise control over sample transport. Due to the extremely small channel dimensions in microfluidic systems, the surface-area-to-volume ratio is significantly increased. Unlike macroscopic flow, where gravity governs interfacial behavior, microfluidic systems are dominated by surface tension and capillary forces. These changes in microscale flow properties, including wall forces, surface tension, wettability, and contact angle, are exhibited in this manner [62].

### 2.3. Theoretical Model and Description Method

Different models corresponding to multiple scales are employed to study microscale flow properties at the microscale. For traditional continuum models analyzing microscale flows (Kn > 0.1), the Navier–Stokes equations are typically applied.

(2) $$\rho \left(\frac{\partial u}{\partial t}+u\cdot \nabla u\right)=-\nabla p+\mu \nabla^{2}u+F$$

Among these parameters, fluid density, velocity vector, pressure, dynamic viscosity, and external forces (e.g., electric and magnetic fields) are considered. However, at the nanoscale (<100 nm), the continuum assumption becomes invalid and requires corrections [63,64].

At the micro/nano scale, fluids no longer fully adhere to the continuum assumption, necessitating molecular-scale simulation methods, such as slip boundary conditions, Knudsen number corrections, and the Boltzmann equation. The Knudsen number (Kn) is defined as presented in Table 1:

(3) $$Kn=\frac{\lambda }{L}$$

**Figure 3:**
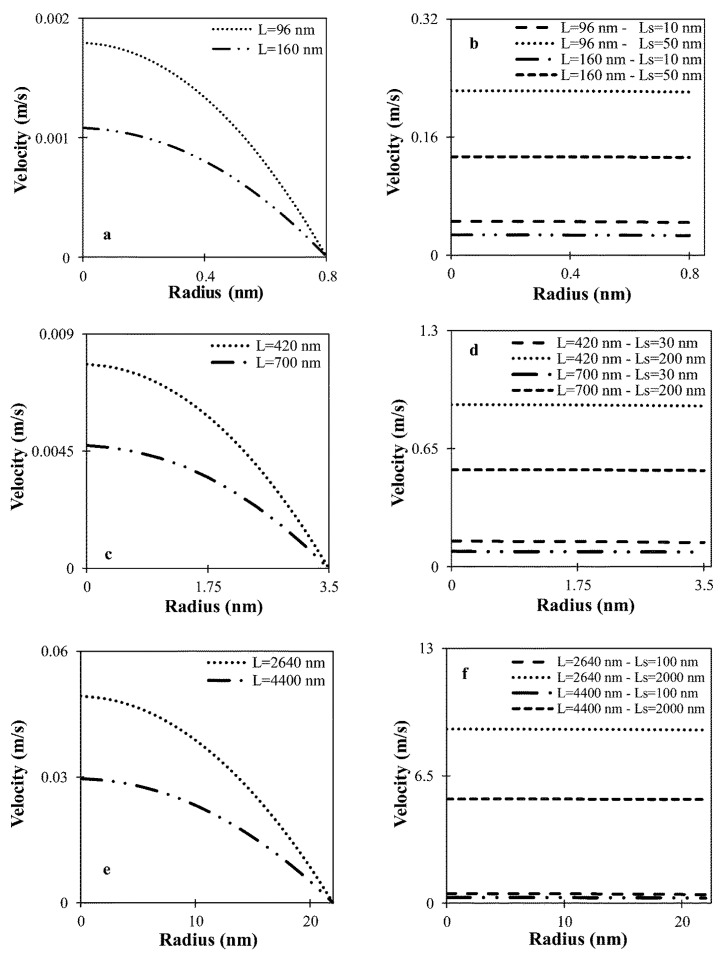
Velocity distribution under different radii and slip lengths (P = 106 Pa, T = 296.15 K): (**a**,**c**,**e**) with no slip conditions; (**b**,**d**,**f**) with slip conditions [65].

Additionally, in nanoscale pores, the viscous effects induced by liquid–solid interactions become significant. Slip velocity is defined as the nonzero fluid velocity at the solid surface; the velocity distribution at various radii and slip lengths is illustrated in Figure 3.

**Figure 4:**
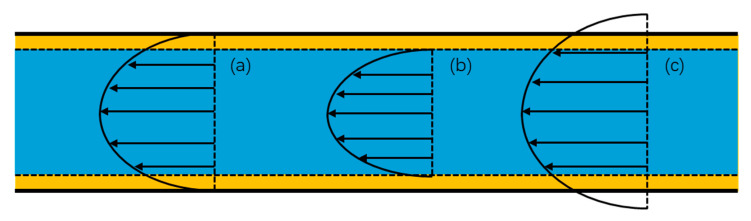
Different types of flow models: (**a**) no slip; (**b**) negative slip; (**c**) positive slip.

Wall conditions are categorized into no-slip, negative slip, and positive slip [66], as depicted in Figure 4. The slip velocity is determined using the following equation:

(4) $$u_{s}=l_{s}\frac{\partial u}{\partial r}$$

Among them $u_{s}$ is the slip velocity, $l_{s}$ is the slip length, and $\frac{\partial u}{\partial r}$ is the velocity gradient [23]. Wall wettability and fluid interactions in microfluidic chips govern the liquid flow behavior within the system.

At the nanoscale (Kn > 0.1), fluid flow under nanoscale conditions is typically governed by viscous forces rather than inertial forces, To account for this effect in confined tube flows, the mass flow rate can be corrected by incorporating $l_{s}$ into the Poiseuille flow expression.

As the fluid channel size shrinks below 100 nm, entering the realm of nanofluidics, liquids can no longer be treated purely as a continuum but rather as assemblies of individual molecules. To accurately capture such molecular-level behaviors, molecular dynamics (MD) simulations are employed, which directly simulate the motion of fluid molecules.

MD directly simulates the motion of fluid molecules and is widely applied to studies on phase states, multiphase pressure, and related phenomena in nanotubes [67,68,69]. Intermolecular forces are computed using the Lennard-Jones potential.

(5) $$U(r)=4\varepsilon \left[{\left(\frac{\sigma }{r}\right)}^{12}-{\left(\frac{\sigma }{r}\right)}^{6}\right]$$

Among them, U(r) is the Lennard-Jones potential energy, r is the intermolecular distance, ε is the maximum position intensity of attraction, and σ is the zero point distance of potential energy.

(6) $${\dot{m}}_{slip}=\frac{\pi r^{4}\rho a_{ext}}{8\mu }\left(1+\frac{4l_{s}}{r}\right)$$

Equation (6) demonstrates that slip significantly enhances the flow rate, especially in ultra-smooth and hydrophobic pores, such as carbon nanotubes (CNTs), where experimental and simulation studies have reported flow rates exceeding the no-slip prediction by over three orders of magnitude [70].

Furthermore, molecular dynamics simulations have shown that the slip length is often shear-rate dependent, particularly in systems with weak wall–fluid interactions or smooth surfaces. Priezjev (2007) proposed the following empirical relationship [71]:

(7) $$L_{s}\propto {\dot{\gamma }}^{\alpha }$$

where ${\dot{\gamma }}^{\alpha }$ is the shear rate, and the exponent α depends on the nature of the surface. For atomically smooth and weakly attractive surfaces, *α* ≈ 1; whereas, for rough or hydrophilic surfaces, the slip becomes nearly independent of $\dot{\gamma }$.

These theoretical insights provide a more complete understanding of fluid transport in nanoscale porous systems and help explain deviations from continuum predictions. They also highlight the importance of accounting for interfacial molecular organization and wall topography when modeling flow in shale or synthetic nanopores.

In addition to molecular dynamics simulations and continuum-level corrections, recent studies have introduced machine learning (ML) techniques as a powerful alternative for predicting nanoscale fluid behaviors such as slip length. Sofos and Karakasidis (2021) developed models using multivariate regression, multilayer perceptron (MLP), and random forest (RF) algorithms, trained on 344 data points collected from both molecular dynamics simulations and the published literature. The slip length was predicted using key features including channel height, surface roughness, wettability, temperature, and wall–fluid interaction strength. Their results showed that nonlinear ML models such as MLP and RF achieved high prediction accuracy (R^2^ > 0.9), outperforming traditional linear regression. Notably, a variable importance analysis revealed that wall–fluid interaction energy and surface roughness contributed over 85% to the model’s predictive power. This demonstrates the potential of ML methods to complement conventional physics-based approaches by efficiently capturing complex interfacial phenomena that influence slip behavior [72].

While MD offers detailed insight at the molecular level, it becomes computationally expensive for larger-scale simulations. To address this, fluid behavior is not adequately described by the Navier–Stokes equations, necessitating the use of mesoscopic and molecular-scale methods. The Boltzmann equation characterizes the velocity distribution function of fluid particles; however, its numerical solution is complex, making the lattice Boltzmann method (LBM) a preferred approach for numerical computations.

(8) $$f_{i}(x+c_{i}\Delta ts,t+\Delta t)=f_{i}(x,t)+\Omega_{i}$$

Among them, $f_{i}$ is the distribution function, $c_{i}$ is the discrete velocity, and $\Omega_{i}$ is the collision operator [73].

These methods together form a multiscale framework that enhances our understanding of transport in nanoporous systems, especially where continuum assumptions break down.

## 3. Experimental Study on Microscopic Seepage Phenomena

Microscopic permeability research primarily focuses on the transport behavior of fluids within porous media at scales ranging from micrometers to nanometers. Owing to the unique characteristics of the microscale, experimental research necessitates high-precision microfluidic technology, advanced visualization techniques, and sophisticated data acquisition and processing methods. The flow characteristics in microscale channels differ significantly from those in macroscopic bulk conditions. To illustrate the experimental exploration of flow behavior across different scales, particularly from nanoconfined pores to microscale fractures, Table 2 compiles a set of representative studies featuring diverse pore structures and fluid systems. This section presents the historical development and current research frontiers of experimental devices and techniques for microscopic seepage studies.

### 3.1. Evolution and Visualization of Microscopic Seepage Experimental Systems

**Figure 5:**
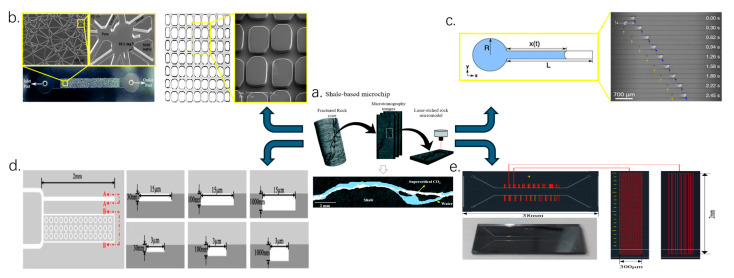
Development of traditional core experiments to modern microfluidic technology. (**a**) Three-dimensional tomography images of real cracks are used to create micromodels [81]. (**b**) Irregular 2D silicon micromodels with dry etching of porous structures are achieved through microfabrication techniques, and 2D PDMS micromodels with regular patterns are established through soft lithography techniques [87,88]. The channel width ranges between 10 and 20 μm. (**c**) Image sequences of capillary flow in open rectangular channels with a width of 200 μm and a depth of 177 μm [89]; (**d**) Single pore channels and porous media channels established by simulating blind end fractures in shale reservoirs [90]. (**e**) The length of the single tube is 2 mm, the width is 3 μm, and the depths are 1000 and 30 nm, respectively. The mixed phase pressure is measured [91].

Microscopic seepage experimental devices have evolved from traditional core experiments to modern microfluidic technology, as illustrated in Figure 5. The core technologies include microfabrication, multiphase flow control, high-precision imaging, and data acquisition, among others.

**Figure 6:**
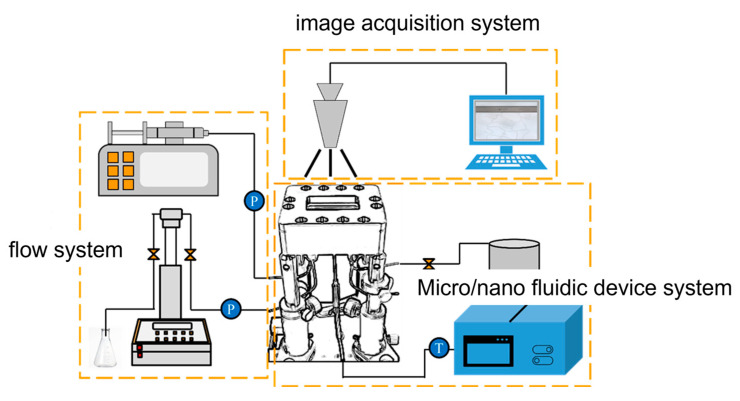
Micro/nano fluidic physical simulation system.

Initially, permeability experiments primarily relied on the Core Flooding System to investigate macroscopic permeability behaviors, including oil–water displacement and gas adsorption. With advancements in microfabrication technology, microfluidic chips have emerged as essential tools for studying microfluidic phenomena. The microfluidic system enables precise regulation of pore geometry and fluid behavior, and is equipped with a thermostatic water bath and temperature sensors for real-time temperature monitoring and control, The complete microfluidic physical simulation setup is illustrated in Figure 6.

**Figure 7:**
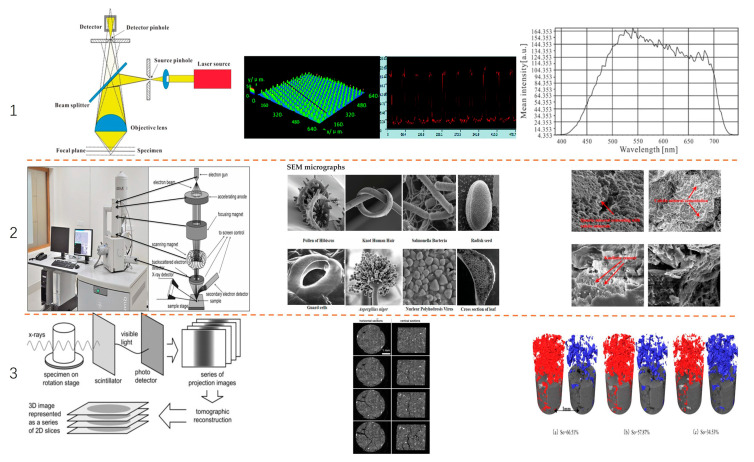
Comparison of visualization systems for three optical imaging technologies, each column including principle diagram, typical output image, and energy application example: (**1**) laser confocal microscope [95,96]; (**2**) scanning electron microscope [97,98]; (**3**) X-ray microscopy CT [99,100].

To visualize fluid flow behavior in micropores, various advanced optical imaging techniques have been developed in recent years for analytical purposes, as illustrated in Figure 7. A laser confocal microscope employs a PDMS soft-lithography microfluidic chip with channel dimensions ranging from 100 to 200 μm. Combined with CLSM + PIV, the 3D velocity field is obtained by scanning layer-by-layer through CLSM. Multiple layer data are stacked to reconstruct the 3D velocity distribution [92]. Environmental scanning electron microscopy (ESEM) enables direct observation of microscale wettability changes. High-resolution scanning transmission electron microscopy (STEM) combined with electron energy loss spectroscopy (EELS) reveals oil–rock interactions and changes in organic layer wettability, influencing fluid transport [93]. Industrial-grade X-ray micro-computed tomography (micro-CT) achieves a resolution of 50–100 μm, utilizing high-speed X-ray scanning to acquire 3D data at different time intervals. Finally, it is combined into a time series to form a dynamic 3D model that can represent microflow properties, such as pore space saturation distribution, fluid flow mechanism, material deformation, and pore scale modeling [94].

### 3.2. Typical Experimental Research Results

The distribution and migration of fluids within porous media are critical to engineering applications including oil and gas extraction, CO_2_ sequestration, and reservoir stimulation. In recent years, substantial advancements have been achieved in the understanding of microscale fluid behavior, attributed to progress in microfluidic experimentation, nanoscale visualization techniques, and multiscale pore network modeling. Meanwhile, the application of artificial intelligence in image enhancement, structure reconstruction, and simulation has introduced novel tools and perspectives that enhance experimental investigations. This section presents representative experimental findings related to phase transition behavior, flow characteristics, scale effects, and pore structure, and discusses their significance for the development of unconventional reservoirs. The phase transition behavior of fluids in nanoscale pores deviates significantly from that observed at macroscopic scales. Ally et al. [76] investigated capillary condensation using high-pressure nanofluidic experiments and determined that pore geometry and wettability play a crucial role in fluid condensation. In addition, Jatukaran et al. [80] conducted sub-10 nm scale visualization experiments to examine evaporation in shale reservoirs, revealing that its occurrence pressure was significantly lower than that predicted by the classical Kelvin equation, indicating that evaporation in nanopores is controlled by the coupling effect of Knudsen diffusion and viscous flow.

In the study of CO_2_ sequestration, Kim et al. [77] employed microfluidic chips to simulate CO_2_ injection into saline aquifers, demonstrating that salt deposition can reduce porosity by 20%, and two deposition modes, bulk crystallization (20–50 μm) and polycrystalline aggregation (10–100 μm), dominate the deposition behavior in different pore size regions. These experimental results indicate that pore size, wettability, and initial saturation state determine the phase transition mode of fluids, which is of great significance for reservoir development and storage optimization.

Recent advances in AI-enhanced micro-CT imaging have significantly enhanced the capability of observing phase transitions at the pore scale. Jackson et al. developed a 3D enhanced deep super-resolution (EDSR) convolutional neural network model, which significantly improved the clarity and continuity of pore-throat structures in low-resolution X-ray CT images. This method facilitates more precise extraction of phase boundaries and enhances the resolution of pore-scale phase distributions, thereby providing high-fidelity input for subsequent phase change experiments and modeling [101].

#### 3.2.1. Fluid Transport Characteristics in Multi Scale Pore Networks

Fluid migration in tight reservoirs, such as shale, is governed by their micro/nano dual-scale pore network. Nguyen et al. [82] applied microfluidic technology to examine the throughput of supercritical CO_2_ (scCO_2_) and N_2_, discovering that scCO_2_ recovery in a connected fracture system reached 90%, much higher than the 40% of N_2_. The main mechanism is the fluid expansion effect driven by gas dissolution precipitation, indicating that CO_2_–oil displacement is not only affected by permeability, but controlled by gas–liquid interactions.

Zhang et al. [83] developed a glass–silicon–glass (GSG) microfluidic model to simulate multiphase flow in shale oil reservoirs. The results indicated that, during water flooding, the fingering phenomenon was prominent, and unstable flow persisted despite increased water viscosity. This study suggests that the geometric structure of microcracks plays a decisive role in fluid transport modes. In addition, Kelly et al. [84] used a dual scale (micro/nano) fluid network to study anomalous diffusion behavior in low-permeability reservoirs, and found that the topological connectivity of the pore network significantly affects the effectiveness of fluid migration.

Wang et al. [75] employed the nanofluidic slim-tube experimental method to investigate the mixed-flow behavior of CO_2_ and hydrocarbons within a multi-scale porous network. The study revealed that, in pores ranging from 100 nm to 10 μm, the CO_2_ diffusion front advances significantly faster than the displacement front, suggesting that molecular diffusion is the dominant mechanism governing nanoscale fluid transport. Furthermore, the study quantified that the minimum miscibility pressure (MMP) in nanopores is lower than conventionally predicted values; whereas, in multi-scale pore structures, MMP exceeds theoretical predictions, highlighting the nonlinear impact of pore size on fluid miscibility. The experiment further demonstrated that CO_2_ mixed flow mitigates the fingering phenomenon induced by fluid viscosity variations, thereby enhancing ultimate oil displacement efficiency.

Ling et al. [86] employed rock-embedded microfluidic chips to investigate the coupled processes of dissolution and permeation in natural rocks using high-resolution optical imaging combined with scanning electron microscopy (SEM) and energy-dispersive spectroscopy (EDS) analysis. The research findings indicate that the heterogeneity of pore structure and mineral composition dictate the spatial distribution of dissolution processes, particularly in shale and carbonate rocks, where acid dissolution induces non-uniform alterations in fracture geometry, thereby influencing fluid transport pathways. Additionally, dynamic imaging technology (with a time resolution of 0.1 s) was utilized to capture real-time changes in microscale fracture interfaces, unveiling the temporal evolution of dissolution–permeation interactions. This study demonstrates that, in tight reservoirs, fluid migration patterns are constrained by the topological characteristics of fracture networks, while mineral composition significantly influences dissolution-driven flow behavior.

Recent studies have demonstrated that artificial intelligence (AI) techniques have significantly enhanced the understanding of flow behavior in multiscale pore systems by facilitating structure reconstruction and flow simulation. Zheng and Zhang [102] developed RockGPT, a generative model that integrates VQ-VAE and GPT for reconstructing 3D digital rocks from single 2D slices. The generated models are able to preserve key morphological and transport properties, thereby enabling high-fidelity flow simulation when full 3D imaging is limited.

Similarly, Fu et al. [103] proposed a stochastic learning method that employs shallow artificial neural networks for the statistical reconstruction of 3D microstructures from 2D slices. The resulting reconstructions successfully retain pore-scale anisotropy and connectivity, both of which are essential for predicting diffusion and fingering phenomena in tight formations. Moreover, as summarized by Delpisheh et al. [104], convolutional neural networks, such as U-Net, have been increasingly adopted for segmenting CT and SEM images, thereby supporting the generation of pore-scale models. Machine learning-based surrogate models have also been incorporated into LBM and PNM workflows to reduce computational costs in simulating flow through complex heterogeneous networks.

#### 3.2.2. The Influence of Nanoscale Effects on Transport Processes

In nanopores, liquid gas phase transitions and confinement effects on molecular transport lead to changes in fluid transport modes. Jatukaran et al. [85] investigated the impact of a 5 nm pore throat on gas transport within a 100 nm pore. The results revealed that small pores created bottlenecks, reducing the gas vaporization rate by a factor of 3000; At low temperatures, multi-component effects further suppress the vaporization rate, with light components preferentially vaporizing while heavy components accumulate in the liquid phase.

In addition, Zhong et al. [74] examined the micro-mechanisms of CO_2_ and N_2_ gas flooding and determined that CO_2_ effectively eliminates the capillary pressure threshold (~2 MPa), enabling stable thin-film displacement. By contrast, N_2_ is unable to effectively displace shale oil due to high capillary pressure, further confirming that nanoscale fluid interactions play a crucial role in determining flow behavior.

To enhance our understanding of nanoscale transport mechanisms, Zhou et al. [105] proposed a physics-informed neural network (PINN) framework for reconstructing the pressure and velocity fields in heterogeneous nanoporous media. The model incorporates governing equations into the loss function and differentiates between organic and inorganic boundaries under varying slip conditions, thereby providing accurate flow predictions, even in sparsely sampled domains.

Artificial neural networks (ANNs) have also been utilized as efficient surrogate models. Alhadri et al. [106] developed an ANN-based surrogate model for simulating hybrid nanofluid thermal flow, which accurately predicted the skin friction coefficient and Nusselt number under varying physical conditions with high accuracy (R^2^ > 0.98). Similarly, Raja et al. [107] employed Bayesian-regularized backpropagation neural networks (BRT-BNNs) to address a three-dimensional hybrid nanofluid flow problem involving radiative and mass fluxes across porous boundaries. The ANN achieved flow and temperature field predictions with mean square errors on the order of 10^−12^, thus offering an effective alternative to traditional numerical solvers in micro-scale modeling. These AI approaches have significantly expanded the scope of nanoscale investigations, particularly when direct observation or full-scale simulation is computationally prohibitive.

#### 3.2.3. The Influence of Pore Structure on Fluid Distribution

The distribution of fluids in reservoirs is governed by pore structure, particularly the impact of the matrix-fracture system on fluid occurrence. Porter et al. [81] investigated the capillary imbibition effect in shale matrices using real-rock microfluidic experiments and determined that water permeability in shale matrices is influenced by fracture width. Larger fractures exhibit a higher initial water absorption rate; however, their final saturation is lower. Capillary imbibition reduces reservoir permeability, thereby influencing oil and gas migration. In addition, Zhang et al. [83] observed in the GSG model experiment that matrix crack structures with varying geometric configurations dictate the distribution patterns of residual oil: oil in microcracks primarily exists as film residue; whereas, in large fractures, it is easily displaced, forming bulk residue. In low-permeability matrices, oil is retained in isolated clusters. This study suggests that optimizing pore structure models can enhance the recovery efficiency of unconventional reservoirs.

To support this optimization process, Ishola and Vilcáez [108] proposed a machine learning framework for predicting permeability from stochastically generated three-dimensional pore structures derived from mercury intrusion capillary pressure (MICP) data. Hundreds of realizations were constructed, sharing equivalent porosity and pore size distributions (PSD), yet exhibiting varying degrees of connectivity. Flow simulations were conducted via finite-volume solvers, while a gradient boosting model was trained using extracted pore-scale features. This surrogate model achieved permeability predictions with a mean absolute error within 10%, while reducing the computational cost by over 150-fold. Such AI-assisted workflows provide a scalable approach for correlating pore geometry with fluid distribution behavior, particularly in heterogeneous rock systems.

### 3.3. Limitations of Experimental Research

Despite significant advancements in studying fluid transport mechanisms in porous media through microscale seepage experiments, several limitations persist. The controlled experimental environment constrains the ability to replicate the complexity of real reservoirs. Although Ling et al. [86] have improved rock-embedded microfluidic technology, experimental conditions still fail to fully replicate the in situ reservoir environment. Furthermore, microscale experiments primarily examine fluid migration behavior at the nanometer-to-micrometer scale; whereas fluid flow in real reservoirs exhibits more intricate multi-scale effects, which requires further exploration of the applicability of experimental results at larger scales. Nguyen et al. [82] also observed that the complexity of fluid interactions exacerbates experimental challenges, including the nonlinear effects of capillary absorption, dissolution precipitation, and heterogeneous flow, among other processes. Variations in experimental setups and medium conditions introduce uncertainties in the results. Although high-resolution imaging techniques, such as X-ray micro-tomography and SEM-EDS, provide detailed pore structure information, achieving full-scale, high-precision tracking of dynamic processes remains challenging (Ling et al. [86]). Additionally, the integration of experimental data with numerical simulations remains inadequate, with many studies still limited to qualitative descriptions. Enhancing the predictive capability of experimental data through numerical simulation remains an area of active research. Thus, future research should focus on optimizing experimental environments, improving the precision of microscale experiments, developing higher-resolution imaging techniques, and integrating numerical simulations with data-driven approaches to enhance the applicability of experimental results and refine microfluidic models for unconventional oil and gas development.

## 4. Numerical Simulation Study on Microscopic Seepage Flow

The development of numerical simulation methods for microscopic seepage has evolved from early simplified geometric models to high-precision computational approaches. In 1949, Purcell introduced the capillary bundle model (BCTM), establishing the theoretical foundation for multiphase flow in capillaries [109]. In 1988, McNamara and Zanetti developed the lattice Boltzmann method (LBM), which established the core numerical framework for direct pore-scale modeling (DPSM), and became a crucial tool for simulating complex pore-scale flows [110]. Alongside LBM, the smooth particle hydrodynamics (SPH) method, introduced by Monaghan in 1992, has emerged as another mainstream technique in DPSM, utilizing meshless particle tracking to model pore-scale flow [111]. In 1998, Øren et al. introduced a digital core-based pore extraction method via pore network modeling (PNM), significantly enhancing computational efficiency [112]. In the 21st century, Piri and Blunt (2005) developed dynamic PNM, incorporating viscous and gravitational effects [113,114], while Raeini et al. (2012) [115] and Jettestuen et al. (2013) [116] improved VoF and level set methods, respectively, to enhance the accuracy of interface tracking. In 2006, MD simulation was employed to analyze equilibrium and transport characteristics of reservoir fluids in oil and gas fields [117], and has since been widely applied to research on fluids limited by nanoporous media [118,119]. In recent years, advancements in artificial intelligence and high-performance computing have driven significant progress in multi-scale coupling and data-driven pore-scale simulations, making them key research frontiers, providing new ideas for solving complex multiphase flow problems. This section will focus on the application and development of LBM and MD in modeling multiphase flow at the pore scale. Compared to the SPH method, which falls under the DPSM framework, LBM holds a more prominent position in microscopic seepage research due to its superior computational efficiency and intrinsic adaptability to porous media.

In this review, we primarily focus on the lattice Boltzmann method (LBM) and molecular dynamics (MD) simulations because they provide complementary strengths for investigating fluid transport at pore- and nano-scales. In our previous work, we systematically reviewed the mechanisms and modeling challenges of nanopore scale oil transport in shale reservoirs [5], where LBM and MD were identified as promising tools for resolving mesoscopic flow behavior and molecular interactions, respectively. Building on these insights, this review highlights how these two methods can be integrated to bridge the scale gap in confined flow modeling. While other methods such as SPH, PNM, and modified Navier–Stokes approaches are briefly discussed, the scope is intentionally centered on LBM and MD due to their strong relevance in multiscale coupling frameworks.

### 4.1. Formation and Improvement of the LBM

The lattice Boltzmann method (LBM) was developed from the lattice gas automata (LGA), initially introduced through the Frisch–Hasslacher–Pomeau (FHP) model. This method simulates fluid motion using Boolean variables and successfully derives the Navier–Stokes equations [120]. However, the LGA is constrained by high statistical noise, strong anisotropy, and complex viscosity calculations, limiting its applicability [121]. To address these limitations, McNamara and Zanetti (1988) proposed simulating the LGA through the Boltzmann equation, replacing Boolean variables with probability distribution functions. This innovation significantly reduced statistical noise and greatly simplified collision calculations by incorporating the Bhatnagar–Gross–Krook (BGK) approximation, thereby enhancing numerical stability and establishing the foundation of the modern LBM [110]. Additionally, the LBM incorporates enhanced lattice structures, such as D2Q9 and D3Q19, to improve isotropy, while the recently developed grayscale LBM has demonstrated superior suitability for fluid simulations in complex porous media [122].

The LBM has also been extensively applied in two-phase flow and interface dynamics simulations. Early studies primarily concentrated on single-phase flow and crystal growth, with the generalized LBM introduced in 2002 for simulating porous media flow [123], Subsequent research in 2004 further extended the LBM applications to supersaturated solutions [124]. In recent years, the LBM has achieved significant advancements in the study of shale gas permeation, microfluidic systems, and solute transport. By 2024, the integration of deep learning methods with the LBM computations is expected to significantly enhance computational efficiency [125]. A representative case of the lattice Boltzmann method (LBM) in simulating fluid flow within microscopic porous media is presented in Table 3. The research encompasses a wide range of complex flow environments, including shale reservoirs, random porous media, limestone formations, CO_2_ geological sequestration sites, and multi-scale pore systems. Various LBM models, such as the multiple-relaxation-time LBM (MRT-LBM), the multiphase complex mixture LBM (MCMP-LBM), the Shan–Chen LBM, and the generalized lattice Boltzmann method, have been employed to model phenomena like multiphase flow, solution-precipitation reactions, phase transitions, and capillary force effects. The accuracy and reliability of these methods were assessed using several validation techniques, including Darcy’s law, Laplace pressure tests, and molecular dynamics (MD) simulations, ensuring the robustness of the simulation outcomes.

#### 4.1.1. Breakthrough of the LBM in Multiphase Flow Research

The LBM has achieved significant advancements in multiphase flow research, particularly in immiscible multiphase flow, wetting effects, and pore-scale solute transport, offering efficient and accurate numerical modeling tools. However, its applicability is constrained by the density ratio issue, posing challenges in modeling high-density two-phase flows, such as CO_2_ injection [123].

Wettability is a critical factor influencing multiphase flow, as it governs the morphology of the oil–water interface and displacement efficiency. Early LBM models exhibited inaccuracies in contact angle calculations. Wetting boundary conditions (WBC) and free energy models (FEM) have been employed to optimize wettability calculations, improve flow prediction accuracy under various wetting conditions, and to expand the LBM applications in reservoir development and capillary displacement processes [135].

Additionally, the LBM has advanced solute transport modeling in porous media by integrating direct numerical simulation (DNS) to analyze solute diffusion, convection, and adsorption at the pore scale, making it particularly suitable for low-permeability reservoirs, shale gas development, and groundwater contamination control [124]. Studies have shown that adsorption at the fluid–solid interface reduces solute migration rates, while small pores tend to retain solutes. The Knudsen effect must be considered to accurately characterize microscopic transport behavior [136].

In summary, the LBM’s advancements in multiphase flow research primarily include the optimization of pseudo-potential multiphase models, improvements in wettability calculations, and multi-scale solute transport simulations, offering novel modeling approaches and numerical methods for oil and gas development, environmental engineering, and microfluidic mechanics [137].

#### 4.1.2. Application of the LBM in Complex Porous Media Seepage

In studies on the application of the LBM in complex porous media seepage, researchers have systematically investigated multiphase flow simulation, CT data-based seepage modeling, the grayscale LBM, and the optimization of heterogeneous porous media, proposing several key improvements.

Guo and Zhao (2002) optimized the SC-LBM to enhance its suitability for complex porous media, thereby improving computational stability [123]. Chen and Doolen (1998) experimentally validated the applicability of the LBM and compared its computational accuracy with the finite volume method (FVM) and the volume fraction method (VOF) in porous media flow simulations [135].

To enhance the applicability of the LBM in real pore structure studies, Kang et al. (2004) proposed a CT data-based LBM seepage modeling approach, utilizing micro-CT scanning technology to directly reconstruct pore structures for seepage simulations [124]. Liu employed high-resolution X-ray CT to scan Berea sandstone samples, generating a three-dimensional pore model via ImageJ (https://imagej.net/ij/, accessed on 4 March 2025), thereby enhancing the accuracy of LBM-based pore-scale permeability studies [132]. Wang et al. investigated the effect of pore wettability on permeability using natural rocks and determined that wettability, capillary number, and oil–water viscosity significantly influence fluid transport pathways and permeability characteristics [138].

Liu and Wu (2016) introduced a pore-scale multiphase flow modeling approach based on Shan–Chen LBM and micro-CT image reconstruction, investigating the effects of fluid wettability and pressure gradient on seepage, thereby validating the applicability of LBM in complex porous media [132]. For multi-scale seepage calculation optimization, in their 2018 study, Zhu and Ma proposed a dual-parameter gray-level Lattice Boltzmann Model capable of independently tuning effective viscosity and permeability. By embedding the Darcy–Brinkman framework, the model improves permeability estimation and enhances the computational efficiency of LBM in simulating complex flows across scales, including shale gas migration, CO_2_ sequestration, and microfluidics [122].

To optimize fluid flow in heterogeneous porous media, Choi et al. reconstructed pore structures using X-ray CT images, introduced an equivalent pore model (PCM), and incorporated the Stokes and Brinkman equations to simulate flow behavior across different pore regions, thereby extending the applicability of the LBM in modeling fluid flow in complex porous media, including rock formations [139]. A single-field fluid–solid coupling method independent of empirical permeability has been introduced, improving the Darcy–Brinkman–Stokes (DBS) model for simulating flow in heterogeneous porous media, thereby enhancing computational efficiency and prediction accuracy [134]. Zhao et al. analyzed flow characteristics in three types of porous media: Berea sandstone, carbonate rock, and shale [137]. Jin investigated solute convection in porous media using direct numerical simulation (DNS), established scaling relationships for various solute transport stages, and examined the influence of pore structure on dissolution flux, thereby providing a computational tool for CO_2_ sequestration and groundwater contamination control [140].

### 4.2. Introduction and Development of MD

The core of the MD (molecular dynamics) method lies in accurately computing microscopic interactions among molecules and atoms within a system. This method is grounded in the principles of classical mechanics, which assert that molecular and atomic motion adheres to Newton’s laws and is highly accurate in systems containing a limited number of molecules and atoms. In MD simulations, the equations of motion are numerically integrated over time to predict subsequent molecular states, thereby enabling dynamic molecular motion simulations. This approach serves as a crucial analytical tool for investigating fluid transport properties—including diffusion rate, viscosity, thermal conductivity, and slip conditions—as well as static characteristics, such as molecular arrangement and radial distribution functions near channel walls. The investigation of fluid transport simulation within nanopores and microscale channels encompasses the behavior of fluids across diverse media, including shale oil, CO_2_–oil interfaces, graphene channels, and silicon nanochannels. This study addresses critical mechanisms, such as shear flow, adsorption layer formation, slip effects, Knudsen diffusion, and water bridge interactions. The simulation methodologies employed encompass non-equilibrium molecular dynamics (NEMD), local average density models (LADM), and lattice Boltzmann methods (LBM). Verification of the results is conducted through NPT calculations, Green–Kubo analysis, Poiseuille flow validation, and potential field comparisons, as detailed in Table 4. Many referenced studies on MD-based transport properties are listed in Table 4 and elaborated in the following subsections.

#### 4.2.1. Nano Confined Fluid Transport Characteristics

It is well established that nanoscale fluid transport behavior deviates significantly from classical fluid mechanics. First, the enhancement of slip effects serves as a primary driving factor. In small-diameter CNTs, weak interactions between water molecules and the wall surface lead to a substantial increase in slip length. For instance, when D = 1.66 nm, the slip length (Ls) is approximately 90 nm. In CNTs with D < 2 nm, water viscosity is reduced by 50% compared to bulk water, further enhancing the slip effect and leading to a flow rate that significantly exceeds predictions based on classical fluid mechanics [32]. Moreover, a substantial reduction in water viscosity within CNTs has been confirmed through Eyring rate theory calculations. Especially in CNTs with D < 1.5 nm, the shear viscosity of water is 50% lower than that of bulk water [151,152]. This viscosity reduction effect is closely related to the molecular structure of water in CNTs. In small-diameter CNTs, water molecules are arranged in a single file mode, and gradually form a layered arrangement when D > 2.0 nm, further affecting their dynamic properties [153]. Within CNTs, the number of hydrogen bonds decreases significantly, reducing intermolecular forces among water molecules and thereby lowering viscosity. Additionally, the activation energy (Ea) of water in CNTs is significantly lower than that of bulk water (0.05 eV vs. 0.6 eV), facilitating molecular migration within CNTs and further reducing viscosity [154]. Furthermore, modifications in density distribution and boundary conditions also contribute to the failure of the HP equation. In CNTs, due to wall interactions, the density of water exhibits periodic fluctuations in both radial and axial directions, rendering the traditional assumption of uniform fluid in the HP equation no longer applicable [26]. Notably, in nanochannels smaller than 9 nm, the combined effects of density stratification and slip phenomena result in a mass flow rate significantly exceeding the HP equation predictions; whereas, in larger-diameter channels, this enhancement effect gradually diminishes [155]. Overall, the ultrafast flow of water in CNTs is the result of the combined effects of nanoscale effects, such as increased slip length, decreased viscosity, reduced activation energy, and changes in density distribution, which significantly render the classical HP equation ineffective in predicting flow within CNTs.

Slip effects and the superlubricity phenomenon are also critical contributors to enhanced fluid flow within nanochannels. Studies have demonstrated that, on hydrophobic surfaces, such as carbon nanotubes (CNTs), weak fluid–wall interactions result in a substantial reduction in shear forces exerted on water molecules near the interface, leading to a considerable increase in slip length and enhancing fluid transport efficiency [156]. However, slip is not an absolute phenomenon, as variations in the interfacial environment may induce a transition of water molecules from a “slip” state to an “adhesion” state. For instance, under high salt concentrations or alterations in surface atomic arrangement, the slip length may decrease or even vanish entirely [157].

The superlubricity phenomenon describes the exceptionally low friction characteristics of fluids, which are further enhanced by nanoscale slip effects. When the channel size is below 9 nm, pronounced fluid density stratification, reduced apparent viscosity, and enhanced slip effects contribute to significantly lower flow resistance than predicted by classical fluid mechanics [155]. However, in highly hydrophilic nanochannels, the opposite effect may occur. Strong interfacial forces may cause the fluid to form a stable retention layer, resulting in a “negative slip effect”, where interfacial fluid movement is restricted, leading to reduced flow capacity. This phenomenon is particularly pronounced in the nanopores of unconventional oil and gas reservoirs, such as shale oil reservoirs [158]. Furthermore, superlubricity in nanolubrication systems is intrinsically linked to slip effects. Under high shear rates, the ordered structure of the lubricant is disrupted, reducing flow resistance and inducing shear thinning behavior, which further enhances slip effects [159].

In summary, viscosity variations of water in CNTs are governed by reductions in hydrogen bonding, activation energy, density stratification, and slip effects. This phenomenon not only influences applications in nanofluidics, drug delivery, and seawater desalination, but facilitates the theoretical refinement and modeling of fluid dynamics in CNTs.

Beyond carbon nanotubes, diverse nanoconfined structures also exhibit varied fluid transport behaviors. For instance, in hydrophilic illite nanopores, water molecules tend to form ordered adsorption layers and structured hydration shells at the solid interface. These interfacial water layers prevent oil molecules from approaching the pore wall, forcing them to migrate axially in a confined core region, thus enhancing their diffusion efficiency [160]. By contrast, graphene nanochannels, due to their atomically smooth and hydrophobic surfaces, enable nearly frictionless water transport, leading to ultrafast flow and extremely large slip lengths beyond classical predictions [161]. Additionally, metal–organic frameworks (MOFs) such as ZIF-8, featuring subnanometer-sized windows and nanocavities, exhibit ultrafast and selective ion transport behavior. This effect arises from partial dehydration of ions as they traverse the angstrom-scale pores, highlighting the importance of size exclusion and framework–fluid interactions in nanoconfined transport. These findings collectively emphasize that the chemical nature, pore geometry, and interfacial interaction of nanoconfinement structures significantly govern transport mechanisms—offering crucial insights for accurately modeling fluid behavior in nanoporous media, such as unconventional oil and gas reservoirs.

#### 4.2.2. Nanofluid Drive Mechanism and Regulation

Nanofluid transport behavior deviates significantly from macroscopic fluid dynamics due to the influence of electric fields, temperature, and pressure, primarily driven by complex interfacial effects, fluid structural changes, and slip phenomena. Among them, the electric field drives the flow, temperature reduces viscosity and induces a thermophoretic effect, and pressure enhances the slip effect and affects the density distribution [162,163,164]. These findings not only expand our understanding of the transport mechanism of nanofluids, but provide theoretical support for technological optimization in fields such as nanofluidics, drug transport, and energy conversion.

## 5. Limitations and Future Perspectives

### 5.1. Limitations

Although significant progress has been made in the study of microscale fluid flow in porous media, several limitations remain that restrict the applicability and accuracy of the current methodologies.

Experimental constraints: Most microfluidic experiments are conducted under idealized laboratory conditions and fail to replicate the high-temperature, high-pressure, and complex chemical environments encountered in subsurface reservoirs. Even advanced rock-embedded chips lack full in situ realism, particularly in terms of mineral heterogeneity and wettability evolution.

Scale transition challenges: A major bottleneck exists in the upscaling of microscale observations to field-relevant parameters. The lack of standardized cross-scale transfer models limits the direct integration of pore-scale results into reservoir-scale simulations, introducing uncertainty in predictive performance.

Computational limitations: While the lattice Boltzmann method (LBM) offers high efficiency and adaptability for porous media, its numerical stability deteriorates in high-density ratio multiphase systems. Conversely, molecular dynamics (MD) simulations offer molecular-level resolution but remain constrained by their intensive computational cost and limited spatial–temporal scales.

Data–model integration gaps: The vast amount of imaging and simulation data is not yet fully leveraged in predictive modeling. Many current applications of artificial intelligence (AI) and machine learning (ML) lack embedded physical principles, which undermines model robustness, especially in extrapolative or multiscale scenarios.

### 5.2. Future Perspectives

To address these challenges and guide future research directions, several promising avenues have been identified.

The development of reservoir-representative experimental platforms. Future microfluidic devices should be capable of operating under in situ reservoir conditions, including elevated temperature and pressure, complex salinity, and multi-phase interactions. The integration of 4D imaging technologies, such as time-resolved micro-CT and X-ray phase contrast imaging, can enable real-time observation of pore-scale fluid dynamics.

The establishment of unified cross-scale modeling frameworks. Coupled modeling approaches that integrate LBM, MD, FEM, and continuum-based CFD models should be further developed. These frameworks should maintain consistent boundary conditions and incorporate scale-aware parameters to bridge microscale transport mechanisms with macroscale flow behavior.

Integration of physics-informed AI models. Moving beyond empirical correlations, future AI applications should incorporate governing equations and physical constraints. Physics-informed neural networks (PINNs), hybrid generative models, and symbolic regression approaches can enhance the interpretability and generalizability of seepage predictions in heterogeneous porous systems.

Application expansion to broader energy and environmental systems. Microscale flow models should be extended to address complex scenarios, such as CO_2_–hydrocarbon phase transitions in nanopores, reactive transport in geothermal reservoirs, and contaminant migration in fractured aquifers. AI-enhanced simulations can support real-time optimization and risk assessment in these systems.

The creation of open-access benchmark datasets and digital pore libraries. Standardized datasets for image-based pore structures, simulation benchmarks, and experimentally validated flow fields will support model calibration, AI training, and inter-method comparison. Community-shared digital rock repositories can accelerate research reproducibility and collaborative development.

In summary, the integration of advanced experimental systems, multiscale modeling techniques, and physics-aware AI approaches represents a promising path forward. These strategies will collectively improve the fidelity, efficiency, and predictive capability of microscale seepage studies and contribute to the sustainable development of subsurface energy systems.

## 6. Conclusions

This review comprehensively summarizes recent advances in the understanding of fluid transport behavior in microporous and nanoporous media, focusing on theoretical frameworks, experimental methodologies, and numerical simulation techniques. At the microscale, seepage processes are governed by surface-dominated mechanisms, such as wettability, interfacial slip, capillary forces, and molecular confinement, which deviate significantly from classical macroscopic flow behavior.

Progress in experimental techniques, including the development of microfluidic devices, advanced imaging systems (e.g., CLSM, SEM, micro-CT), and nanofluidic visualization platforms, has greatly enhanced our ability to observe phase transitions and transport behavior at fine spatial and temporal resolutions. Meanwhile, numerical methods such as the lattice Boltzmann method (LBM) and molecular dynamics (MD) have proven effective for simulating multiphase transport and fluid–solid interactions at the pore and molecular scales.

Importantly, the integration of multi-scale modeling strategies and AI techniques—including deep learning, surrogate modeling, and generative reconstruction—has begun to reshape microscale seepage research. These hybrid approaches enable more accurate, efficient, and physically consistent predictions, marking a transformative shift in the field.

By bridging experimental observations, numerical simulations, and data-driven modeling, this review provides a unified understanding of microscopic flow behavior in porous systems. The findings lay the foundation for future developments in unconventional reservoir characterization, CO_2_ storage optimization, and smart fluid transport systems in energy and environmental applications.

## Figures and Tables

**Table 1:** Applicable models for different Kn values.

Kn Value Range	Applicable Models
Kn < 0.001	Application of Navier–Stokes equations (for continuous medium flow)
0.001 < Kn < 0.1	Slip flow needs to be corrected
0.1 < Kn < 10	Transition flow, Navier–Stokes failure
Kn > 10	Free molecular flow requires molecular dynamics (MD)

**Table 2:** Conclusion of microscale flow properties under different experimental conditions.

Scale of Experiment	Fluid Type	Research Content	References
10^6^ nanopores, pore size 60 nm, microchannel connection (200 μm)	Light crude oil (West Texas Crude), N_2_, CO_2_	CO_2_, N_2_ enhanced oil recovery	[74]
Glass–silicon–glass structure, pore size 100 nm–10 μm	CO_2_, n-heptane, pentane–dodecane mixture	CO_2_ miscibility pressure	[75]
Microfluidic chip with 150 nm SiO_2_ particles in ordered/disordered packing	Propane (C_3_H_8_), CO_2_	CO_2_, propane capillary condensation pressure	[76]
Single straight-through channel with pore diameter of 250 μm, random pore structure with pore diameter 50–400 μm	CO_2_, high-salinity brine (4.7 mM NaCl)	CO_2_ injection efficiency and salt precipitation relationship	[77]
Channel depth 88 nm, width 7 μm	Propane (C_3_H_8_)	Bubble growth dominant conditions	[78]
20 parallel nanofluidic channels (depth 50 nm, width 5 μm)	Hexane, heptane, octane	Bubble nucleation temperature under 50 nm confinement	[79]
Pore depth 9 nm, width 225 nm	Propane (C_3_H_8_)	Evaporation pressure	[80]
Shale micromodel with pore width 50–400 μm	n-Decane, supercritical CO_2_, brine	Capillary effect and matrix wettability relationship; fracture roughness and CO_2_ migration resistance	[81]
Fracture width 400–1000 μm, depth 500 μm	n-Decane, supercritical CO_2_, N_2_	Comparison of CO_2_ and N_2_ huff-n-puff recovery efficiency	[82]
Matrix pores 3–10 μm, microfractures 100–200 μm	n-Decane, deionized water	Residual oil distribution	[83]
Nanopores 200 nm–5 μm, microfractures 10–100 μm	Deionized water, isopropanol, fluorescent-labeled oil phase	Flow behavior in microfractures and nanopores	[84]
100 nm macropores, 5 nm throat	Methane–propane–pentane mixture (10%/40%/50%)	Light and heavy hydrocarbon gas release rate under 5 nm throat conditions	[85]
Pore size 0.1–1 μm, fracture width 1–1000 μm	1% HCl, CO_2_ gas	Differences in fracture and pore structure between high and low carbonate rocks	[86]

**Table 3:** Overview of the Lattice Boltzmann Method (LBM) Applications in Porous Media Flow Simulations.

Research Object	LBM Model	Validation Method	References
Shale/tight oil reservoir	Multiple relaxation time lattice Boltzmann method (MRT-LBM), considering fluid–fluid and fluid–surface interaction forces	1. Fick’s second law calibration for diffusion; 2. Molecular dynamics (MD) calibration for adsorption; 3. MD calibration for miscible flow rate	[126]
Random porous media	Multicomponent multiphase LBM (MCMP-LBM), Shan–Chen interaction force model	1. Droplet contact angle validation; 2. Reaction–diffusion experiment comparison; 3. Fracture acidizing simulation	[127]
Indiana limestone	MRT-LBM combined with μ-CT data, permeability calculation using Darcy’s Law	1. REV analysis to determine the minimum representative volume; 2. Pore-permeability log-normal distribution validation	[128]
Dissolution–precipitation coupled reactive flow in porous media, CO_2_ geological sequestration	Multicomponent multiphase LBM (MCMP-LBM) coupled with heat-mass transfer model	1. Poiseuille flow validation; 2. Natural convection experiment comparison; 3. Grid independence analysis	[129]
Shale oil–CO_2_–water multiphase flow behavior, CO_2_ huff-n-puff process	Improved MCMP-LBM, considering competitive adsorption and interfacial tension	1. Laplace pressure test; 2. Contact angle calibration; 3. MD calibration for diffusion	[130]
Application of LBM in multiphase multicomponent flow simulation, phase change, and interfacial tension	Shan–Chen type multiphase LBM, introducing non-ideal gas state equation	1. Laplace test; 2. Phase separation simulation; 3. Diffusion coefficient validation	[131]
Immiscible two-phase flow in complex porous media	Shan–Chen multiphase LBM, combined with micro-CT reconstructed pore structure	1. Laplace pressure validation; 2. Wettability experiment comparison; 3. FVM-VOF comparison	[132]
Multiscale porous media flow, improved gray LBM	Two-parameter gray LBM, independently controlling effective viscosity and permeability	1. Darcy–Brinkman equation validation; 2. Velocity profile analysis; 3. Isotropy test	[122]
Multiscale heterogeneous material liquid–vapor dynamics	Shan–Chen LBM combined with WBS partial bounce-back method	1. Maxwell construction validation; 2. Laplace law test; 3. Complex pore experiment comparison	[133]
Multiscale transport in heterogeneous porous media	Single-field coupling model, implicitly describing effective parameters for solid influence	1. DBS method comparison; 2. Scalar transport validation; 3. Isotropy test	[134]

**Table 4:** Development and Validation of a Multiscale Molecular Simulation Model for Fluid Transport in Nanopores.

Simulation Scale	Model and Research Object	Validation	References
Width W = 5~10σ, NEMD 10^7^ steps	Local average density model (LADM) combined with hard-rod weighting function to study shear flow of fluids in confined pores	NEMD computes shear viscosity, Green–Kubo method calculates local viscosity, error < 5%.	[141]
Illite pore diameter 8 nm, shale oil simulation 8 ns	Multicomponent shale oil adsorption layer model to study shale oil adsorption in nanoscale pores	Adsorption layer thickness computed and compared with experiments, 20 MPa methane/octane NPT calculation error 2.47%.	[142]
Quartz pore 8 nm, CO_2_ 50% proportion	CO_2_–oil interface friction reduction model to study CO_2_ in aqueous film environments	NPT computes octane density, compared with NIST data (0.69 g/cm^3^), error < 3%.	[143]
Graphene channel 50 × 25 × 90 Å^3^	Water film-enhanced oil transport model to study the impact of water on oil transport in graphene nanochannels	Compute oil–water interface potential energy distribution, radial distribution function (RDF) analyzes oil–water molecular interaction.	[144]
Pore diameter 2 nm~1 mm	Slip/adsorption/surface diffusion transport mechanism study of shale gas transport in micro/nanopores across multiple scales	LBM simulation of apparent permeability, Knudsen number comparison error < 5%.	[145]
Pore diameter 2–10 nm, NEMD 2 ns	Study of interfacial and viscous resistance effects on the flow behavior of n-alkanes in nanopores	Field validation (NIST data error < 3%), slip length analysis of different pore flow rate changes.	[146]
Channel height 5.4, 54 nm	Knudsen number-dependent transport model to study gas transport in nanochannels	MD computes velocity distribution, DSMC computes Knudsen minimum point location, error < 3%.	[147]
Simulation box 18 × 10.5 × 10.5σ	Shear stress and viscosity decomposition model to study local shear viscosity in non-uniform fluids	Shear rate verification of velocity distribution, Green–Kubo computes viscosity, compared with experiments.	[148]
40 Å wide silicon nanochannel, 32.28 × 73.90 × 40.00 Å^3^	Einstein relation (MSD) + Green–Kubo formula to calculate diffusion coefficient in silicon nanochannels	MSD and Green–Kubo method calculation of diffusion coefficients are consistent.	[149]
Pore size 5, 10, 15 nm	P-H (potassium–hydroxyl structure) and H-H (hydroxyl–hydroxyl structure) pore water bridge formation mechanism to study the role of water bridges in clay nanopores	Compute water bridge interfacial potential energy, PMF calculates hydrocarbon adhesion potential under different pore sizes.	[150]

## Data Availability

No new data were created or analyzed in this study. Data sharing is not applicable to this article.

## References

[1] Li L., Zhang D., Su Y., Zhang X., Lu M., Wang H. (2024). Microfluidic Insights into CO_2_ Sequestration and Enhanced Oil Recovery in Laminated Shale Reservoirs: Post-Fracturing Interface Dynamics and Micro-Scale Mechanisms. Adv. Geo-Energy Res..

[2] Han B., Wang S., Zhang Z., Wang Y. (2024). Numerical Simulation of Geothermal Reservoir Thermal Recovery of Heterogeneous Discrete Fracture Network-Rock Matrix System. Energy.

[3] Jangda Z., Menke H., Busch A., Geiger S., Bultreys T., Lewis H., Singh K. (2023). Pore-Scale Visualization of Hydrogen Storage in a Sandstone at Subsurface Pressure and Temperature Conditions: Trapping, Dissolution and Wettability. J. Colloid Interface Sci..

[4] Hou Y., Deng H., Pan F., Chen W., Du Q., Jiao K. (2019). Pore-Scale Investigation of Catalyst Layer Ingredient and Structure Effect in Proton Exchange Membrane Fuel Cell. Appl. Energy.

[5] Chang Y., Yang Z., Zhang Y., Niu Z., Chen X. (2023). Micro-Nano Scale Confined Flow Characteristics and Mechanism in Tight Reservoir: Insights from Experimental and Molecular Simulation Studies. Geoenergy Sci. Eng..

[6] Ranjbarzadeh R., Sappa G. (2025). Numerical and Experimental Study of Fluid Flow and Heat Transfer in Porous Media: A Review Article. Energies.

[7] Fani M., Pourafshary P., Mostaghimi P., Mosavat N. (2022). Application of Microfluidics in Chemical Enhanced Oil Recovery: A Review. Fuel.

[8] Bao B., Zhao S. (2021). A Review of Experimental Nanofluidic Studies on Shale Fluid Phase and Transport Behaviors. J. Nat. Gas Sci. Eng..

[9] Cai J., Qin X., Xia X., Jiao X., Chen H., Wang H., Xia Y. (2024). Numerical Modeling of Multiphase Flow in Porous Media Considering Micro- and Nanoscale Effects: A Comprehensive Review. Gas Sci. Eng..

[10] Liehui Z., Baochao S., Yulong Z., Zhaoli G. (2019). Review of Micro Seepage Mechanisms in Shale Gas Reservoirs. Int. J. Heat Mass Transf..

[11] Yin Y., Cui Y., Jing L. (2024). Clogging and Unclogging of Fine Particles in Porous Media: Micromechanical Insights from an Analog Pore System. WATER Resour. Res..

[12] Forouzandeh F., Zhu X., Alfadhel A., Ding B., Walton J.P., Cormier D., Frisina R.D., Borkholder D.A. (2019). A Nanoliter Resolution Implantable Micropump for Murine Inner Ear Drug Delivery. J. Control. Release.

[13] Jiang X., He G., Cai J., Xiao W., Hussain C., Mishra A. (2018). Microscale Flow and Separation Process Analysis in the Nanoporous Crystal Layer. New Polymer Nanocomposites for Environmental Remediation.

[14] Foroozesh J., Kumar S. (2020). Nanoparticles Behaviors in Porous Media: Application to Enhanced Oil Recovery. J. Mol. Liq..

[15] Pengfei J., Pengwan W., Shangwen Z., Huaichang W., Xiangyang C. (2022). Study on the Microscopic Pore Structures of Coal Measure Reservoirs in the Shanxi Formation, Eastern Ordos Basin. Front. Earth Sci..

[16] Lai J., Wang G., Wang Z., Chen J., Pang X., Wang S., Zhou Z., He Z., Qin Z., Fan X. (2018). A Review on Pore Structure Characterization in Tight Sandstones. Earth-Sci. Rev..

[17] Liu X., Wang J., Ge L., Hu F., Li C., Li X., Yu J., Xu H., Lu S., Xue Q. (2017). Pore-Scale Characterization of Tight Sandstone in Yanchang Formation Ordos Basin China Using Micro-CT and SEM Imaging from Nm-to Cm-Scale. Fuel.

[18] Tian W., Lu S., Huang W., Wang S., Gao Y., Wang W., Li J., Xu J., Zhan Z. (2019). Study on the Full-Range Pore Size Distribution and the Movable Oil Distribution in Glutenite. Energy Fuels.

[19] Bakhshian S., Hosseini S.A., Shokri N. (2019). Pore-Scale Characteristics of Multiphase Flow in Heterogeneous Porous Media Using the Lattice Boltzmann Method. Sci. Rep..

[20] Zhang T., Hu Q., Tian Q., Ke Y., Wang Q. (2024). Small Angle Neutron Scattering Studies of Shale Oil Occurrence Status at Nanopores. Adv. Geo-Energy Res..

[21] Neto C., Evans D.R., Bonaccurso E., Butt H.-J., Craig V.S. (2005). Boundary Slip in Newtonian Liquids: A Review of Experimental Studies. Rep. Prog. Phys..

[22] Qi D., Hoelzle D.J., Rowat A.C. (2012). Probing Single Cells Using Flow in Microfluidic Devices. Eur. Phys. J. Spec. Top..

[23] Stokes G.G. (2007). On the Theories of the Internal Friction of Fluids in Motion, and of the Equilibrium and Motion of Elastic Solids. Classics of Elastic Wave Theory.

[24] Navier C. (1822). Mémoire Sur Les Lois Du Mouvement Des Fluides.

[25] Huang D.M., Sendner C., Horinek D., Netz R.R., Bocquet L. (2008). Water Slippage versus Contact Angle: A Quasiuniversal Relationship. Phys. Rev. Lett..

[26] Karim K.E., Kim B. (2021). First Law of Thermodynamics on the Boundary for Flow through a Carbon Nanotube. Phys. Rev. E.

[27] Zhang R.L., Du G.J., Wang M.F., Li S.Y. (2022). Controlling Water Flow in Pattern-Charged Nanotubes. J. Nano Res..

[28] Wei N., Peng X., Xu Z. (2014). Breakdown of Fast Water Transport in Graphene Oxides. Phys. Rev. E.

[29] Zhang X., Ma F., Yin S., Wallace C.D., Soltanian M.R., Dai Z., Ritzi R.W., Ma Z., Zhan C., Lü X. (2021). Application of Upscaling Methods for Fluid Flow and Mass Transport in Multi-Scale Heterogeneous Media: A Critical Review. Appl. Energy.

[30] Tomo Y., Askounis A., Ikuta T., Takata Y., Sefiane K., Takahashi K. (2018). Superstable Ultrathin Water Film Confined in a Hydrophilized Carbon Nanotube. Nano Lett..

[31] Thomas J.A., McGaughey A.J., Kuter-Arnebeck O. (2010). Pressure-Driven Water Flow through Carbon Nanotubes: Insights from Molecular Dynamics Simulation. Int. J. Therm. Sci..

[32] Thomas J.A., McGaughey A.J. (2008). Reassessing Fast Water Transport through Carbon Nanotubes. Nano Lett..

[33] Holt J.K. (2008). Methods for Probing Water at the Nanoscale. Microfluid. Nanofluid..

[34] Hummer G., Rasaiah J., Noworyta J. (2001). Nanoscale Hydrodynamics: Enhanced Flow in Carbon Nanotubes. Nature.

[35] Jing W., Zhang L., Li A., Liu T., Cheng Y., Sun H., Yang Y., Zhu G., Yao J., Zhong J. (2024). Phase Behaviors of Gas Condensate at Pore Scale: Direct Visualization via Microfluidics and in-Situ CT Scanning. SPE J..

[36] Tang M., Zhang T., Ma Y., Hao D., Yang X., Li Y. (2023). Experimental Study on Fracture Effect on the Multiphase Flow in Ultra-Low Permeability Sandstone Based on LF-NMR. Geoenergy Sci. Eng..

[37] Gogoi S., Gogoi S.B. (2019). Review on Microfluidic Studies for EOR Application. J. Pet. Explor. Prod. Technol..

[38] Anbari A., Chien H., Datta S.S., Deng W., Weitz D.A., Fan J. (2018). Microfluidic Model Porous Media: Fabrication and Applications. Small.

[39] Mostowfi F., Molla S., Tabeling P. (2012). Determining Phase Diagrams of Gas–Liquid Systems Using a Microfluidic PVT. Lab A Chip.

[40] Zheng B., Tice J.D., Roach L.S., Ismagilov R.F. (2004). A Droplet-based, Composite PDMS/Glass Capillary Microfluidic System for Evaluating Protein Crystallization Conditions by Microbatch and Vapor-diffusion Methods with On-chip X-ray Diffraction. Angew. Chem. Int. Ed..

[41] Verma N., Pandya A. (2022). Challenges and Opportunities in Micro/Nanofluidic and Lab-on-a-Chip. Prog. Mol. Biol. Transl. Sci..

[42] Rodríguez C.F., Andrade-Pérez V., Vargas M.C., Mantilla-Orozco A., Osma J.F., Reyes L.H., Cruz J.C. (2023). Breaking the Clean Room Barrier: Exploring Low-Cost Alternatives for Microfluidic Devices. Front. Bioeng. Biotechnol..

[43] Cobos S., Carvalho M., Alvarado V. (2009). Flow of Oil-Water Emulsions through a Constricted Capillary. Int. J. Multiph. FLOW.

[44] Wang H., Wang E., Liu Z., Gao D., Yuan R., Sun L., Zhu Y. (2015). A Novel Carbon Nanotubes Reinforced Superhydrophobic and Superoleophilic Polyurethane Sponge for Selective Oil-Water Separation through a Chemical Fabrication. J. Mater. Chem. A.

[45] Gong D., Grimes C.A., Varghese O.K., Hu W., Singh R.S., Chen Z., Dickey E.C. (2001). Titanium Oxide Nanotube Arrays Prepared by Anodic Oxidation. J. Mater. Res..

[46] Fuquan S., Xiao H., Genmin Z., Weiyao Z. (2018). The Characteristics of Water Flow Displaced by Gas in Nano Arrays. Chin. J. Theor. Appl. Mech..

[47] Wegner J., Ganzer L. (2017). Rock-on-a-Chip Devices for High p, T Conditions and Wettability Control for the Screening of EOR Chemicals.

[48] Lee H., Lee S.G., Doyle P.S. (2015). Photopatterned Oil-Reservoir Micromodels with Tailored Wetting Properties. Lab A Chip.

[49] Zhang Y.Q., Sanati-Nezhad A., Hejazi S.H. (2018). Geo-Material Surface Modification of Microchips Using Layer-by-Layer (LbL) Assembly for Subsurface Energy and Environmental Applications. Lab A Chip.

[50] Wang H., Su Y., Wang W., Li G., Zhang Q. (2023). Simulation on Liquid Flow in Shale Nanoporous Media Based on Lattice Boltzmann Method. Acta Pet. Sin..

[51] Zheng J., Ju Y., Wang M. (2018). Pore-scale Modeling of Spontaneous Imbibition Behavior in a Complex Shale Porous Structure by Pseudopotential Lattice Boltzmann Method. J. Geophys. Res. Solid Earth.

[52] Zhang J., Song H., Zhu W., Wang J. (2021). Liquid Transport through Nanoscale Porous Media with Strong Wettability. Transp. Porous Media.

[53] Wu S., Li Z., Zhang C., Lv G., Zhou P. (2021). Nanohydrodynamic Model and Transport Mechanisms of Tight Oil Confined in Nanopores Considering Liquid–Solid Molecular Interaction Effect. Ind. Eng. Chem. Res..

[54] Xing L., Zhang Y. (2024). A Many-Body Dissipative Particle Dynamics Simulation of Flow Performance in Capillary Channel. Comput. Mater. Sci..

[55] Sasaki K., Ishiwatari Y., Ueno K., Kojima T., Banno T., Arai N. (2024). Molecular Modelling of Active Oil Droplet Propulsion: Insights from Dissipative Particle Dynamics Simulation. Chem. Phys. Lett..

[56] Musharaf H.M., Roshan U., Mudugamuwa A., Trinh Q.T., Zhang J., Nguyen N.-T. (2024). Computational Fluid-Structure Interaction in Microfluidics. Micromachines.

[57] Yu H., Xu H., Fan J., Zhu Y.-B., Wang F., Wu H. (2020). Transport of Shale Gas in Microporous/Nanoporous Media: Molecular to Pore-Scale Simulations. Energy Fuels.

[58] Kavokine N., Netz R.R., Bocquet L. (2021). Fluids at the Nanoscale: From Continuum to Subcontinuum Transport. Annu. Rev. Fluid Mech..

[59] Han W., Li W., Zhang H. (2024). A Comprehensive Review on the Fundamental Principles, Innovative Designs, and Multidisciplinary Applications of Micromixers. Phys. Fluids.

[60] Wang L., Fan J. (2010). Nanofluids Research: Key Issues. Nanoscale Res. Lett..

[61] Reynolds O. (1883). XXIX. An Experimental Investigation of the Circumstances Which Determine Whether the Motion of Water Shall Be Direct or Sinuous, and of the Law of Resistance in Parallel Channels. Philos. Trans. R. Soc. Lond..

[62] Qin X., Wu J., Xia Y., Wang H., Cai J. (2024). Multicomponent Image-Based Modeling of Water Flow in Heterogeneous Wet Shale Nanopores. Energy.

[63] Batchelor G.K. (2000). An Introduction to Fluid Dynamics.

[64] Kundu P.K., Cohen I.M., Dowling D.R., Capecelatro J. (2024). Fluid Mechanics.

[65] Jamali J., Shoghl S.N. (2014). Computational Fluid Dynamics Modeling of Fluid Flow and Heat Transfer in the Central Pore of Carbon Nanopipes. RSC Adv..

[66] Wu K., Chen Z., Li J., Li X., Xu J., Dong X. (2017). Wettability Effect on Nanoconfined Water Flow. Proc. Natl. Acad. Sci. USA.

[67] Allen M.P., Tildesley D.J. (2017). Computer Simulation of Liquids.

[68] Tian Y., Ju B., Wang X., Wang H., Hu J., Huang Y., Liu N., Dong Y. (2021). Study on Phase Behavior of CO_2_/Hydrocarbons in Shale Reservoirs Considering Sieving Effect and Capillary Pressure. Nat. Resour. Res..

[69] Feng Q., Xu S., Xing X., Zhang W., Wang S. (2020). Advances and Challenges in Shale Oil Development: A Critical Review. Adv. Geo-Energy Res..

[70] Joseph S., Aluru N.R. (2008). Why Are Carbon Nanotubes Fast Transporters of Water?. Nano Lett..

[71] Priezjev N.V. (2007). Effect of Surface Roughness on Rate-Dependent Slip in Simple Fluids. J. Chem. Phys..

[72] Sofos F., Karakasidis T.E. (2021). Nanoscale Slip Length Prediction with Machine Learning Tools. Sci. Rep..

[73] Succi S. (2001). The Lattice Boltzmann Equation for Fluid Dynamics and Beyond.

[74] Zhong J., Abedini A., Xu L., Xu Y., Qi Z., Mostowfi F., Sinton D. (2018). Nanomodel Visualization of Fluid Injections in Tight Formations. Nanoscale.

[75] Wang Z., Zhang T., Liu S., Ding K., Liu T., Yao J., Sun H., Yang Y., Zhang L., Wang W. (2024). Unveiling Nanoscale Fluid Miscible Behaviors with Nanofluidic Slim-Tube. Energy Environ. Sci..

[76] Ally J., Molla S., Mostowfi F. (2016). Condensation in Nanoporous Packed Beds. Langmuir.

[77] Kim M., Sell A., Sinton D. (2013). Aquifer-on-a-Chip: Understanding Pore-Scale Salt Precipitation Dynamics during CO_2_ Sequestration. Lab A Chip.

[78] Bao B., Zandavi S.H., Li H., Zhong J., Jatukaran A., Mostowfi F., Sinton D. (2017). Bubble Nucleation and Growth in Nanochannels. Phys. Chem. Chem. Phys..

[79] Alfi M., Nasrabadi H., Banerjee D. (2016). Experimental Investigation of Confinement Effect on Phase Behavior of Hexane, Heptane and Octane Using Lab-on-a-Chip Technology. Fluid Phase Equilibria.

[80] Jatukaran A., Zhong J., Persad A.H., Xu Y., Mostowfi F., Sinton D. (2018). Direct Visualization of Evaporation in a Two-Dimensional Nanoporous Model for Unconventional Natural Gas. ACS Appl. Nano Mater..

[81] Porter M.L., Jiménez-Martínez J., Martinez R., McCulloch Q., Carey J.W., Viswanathan H.S. (2015). Geo-Material Microfluidics at Reservoir Conditions for Subsurface Energy Resource Applications. Lab A Chip.

[82] Nguyen P., Carey J.W., Viswanathan H.S., Porter M. (2018). Effectiveness of Supercritical-CO_2_ and N_2_ Huff-and-Puff Methods of Enhanced Oil Recovery in Shale Fracture Networks Using Microfluidic Experiments. Appl. Energy.

[83] Zhang Y., Zhou C., Qu C., Wei M., He X., Bai B. (2019). Fabrication and Verification of a Glass–Silicon–Glass Micro-/Nanofluidic Model for Investigating Multi-Phase Flow in Shale-like Unconventional Dual-Porosity Tight Porous Media. Lab Chip.

[84] Kelly S.A., Torres-Verdín C., Balhoff M.T. (2016). Subsurface to Substrate: Dual-Scale Micro/Nanofluidic Networks for Investigating Transport Anomalies in Tight Porous Media. Lab A Chip.

[85] Jatukaran A., Zhong J., Abedini A., Sherbatian A., Zhao Y., Jin Z., Mostowfi F., Sinton D. (2019). Natural Gas Vaporization in a Nanoscale Throat Connected Model of Shale: Multi-Scale, Multi-Component and Multi-Phase. Lab A Chip.

[86] Ling B., Sodwatana M., Kohli A., Ross C.M., Jew A., Kovscek A.R., Battiato I. (2022). Probing Multiscale Dissolution Dynamics in Natural Rocks through Microfluidics and Compositional Analysis. Proc. Natl. Acad. Sci. USA.

[87] Gunda N.S.K., Bera B., Karadimitriou N.K., Mitra S.K., Hassanizadeh S.M. (2011). Reservoir-on-a-Chip (ROC): A New Paradigm in Reservoir Engineering. Lab A Chip.

[88] Auset M., Keller A.A. (2004). Pore-scale Processes That Control Dispersion of Colloids in Saturated Porous Media. Water Resour. Res..

[89] Kolliopoulos P., Jochem K.S., Lade R.K., Francis L.F., Kumar S. (2019). Capillary Flow with Evaporation in Open Rectangular Microchannels. Langmuir.

[90] Wang Y., Lei Z., Sun L., Pan X., Liu Y., Xu Z., Zheng X., Wang Y., Liu P. (2024). Study on the Minimum Miscibility Pressure and Phase Behavior of CO2–Shale Oil in Nanopores. Chem. Eng. J..

[91] Pan X., Sun L., Chen F., Huo X., Wang Y., Feng C., Zheng X., Zhang Z. (2024). Minimum Miscibility Pressure of the CO_2_-Hydrocarbon System Based on Nanofluidics. Energy Fuels.

[92] de Winter D.A.M., Weishaupt K., Scheller S., Frey S., Raoof A., Hassanizadeh S.M., Helmig R. (2021). The Complexity of Porous Media Flow Characterized in a Microfluidic Model Based on Confocal Laser Scanning Microscopy and Micro-PIV. Transp. Porous Media.

[93] Ivanova A., Mitiurev N., Cheremisin A., Orekhov A., Kamyshinsky R., Vasiliev A. (2019). Characterization of Organic Layer in Oil Carbonate Reservoir Rocks and Its Effect on Microscale Wetting Properties. Sci. Rep..

[94] Jahanbakhsh A., Wlodarczyk K.L., Hand D.P., Maier R.R., Maroto-Valer M.M. (2020). Review of Microfluidic Devices and Imaging Techniques for Fluid Flow Study in Porous Geomaterials. Sensors.

[95] Teng X., Li F., Lu C. (2020). Visualization of Materials Using the Confocal Laser Scanning Microscopy Technique. Chem. Soc. Rev..

[96] Cui S.S., Li Z.A., Sun X.D., Liu X.B. (2021). Laser Confocal Scanning Microscope Analysis on Micro-Pore Structures and Occurrence State of Organic Matter in Tight Rocks.

[97] Rathnaweera T.D., Ranjith P.G., Perera M.S.A., Haque A., Lashin A., Al Arifi N., Chandrasekharam D., Yang S.-Q., Xu T., Wang S.H. (2015). CO_2_-Induced Mechanical Behaviour of Hawkesbury Sandstone in the Gosford Basin: An Experimental Study. Mater. Sci. Eng. A.

[98] Kannan M. (2018). Scanning Electron Microscopy: Principle, Components and Applications. A Textbook on Fundamentals and Applications of Nanotechnology.

[99] Li J., Jiang H., Wang C., Zhao Y., Gao Y., Pei Y., Wang C., Dong H. (2017). Pore-Scale Investigation of Microscopic Remaining Oil Variation Characteristics in Water-Wet Sandstone Using CT Scanning. J. Nat. Gas Sci. Eng..

[100] Landis E.N., Keane D.T. (2010). X-Ray Microtomography. Mater. Charact..

[101] Jackson S.J., Niu Y., Manoorkar S., Mostaghimi P., Armstrong R.T. (2022). Deep Learning of Multiresolution X-Ray Micro-Computed-Tomography Images for Multiscale Modeling. Phys. Rev. Appl..

[102] Zheng Q., Zhang D. (2022). RockGPT: Reconstructing Three-Dimensional Digital Rocks from Single Two-Dimensional Slice with Deep Learning. Comput. Geosci..

[103] Fu J., Xiao D., Li D., Thomas H.R., Li C. (2022). Stochastic Reconstruction of 3D Microstructures from 2D Cross-Sectional Images Using Machine Learning-Based Characterization. Comput. Methods Appl. Mech. Eng..

[104] Delpisheh M., Ebrahimpour B., Fattahi A., Siavashi M., Mir H., Mashhadimoslem H., Abdol M.A., Ghorbani M., Shokri J., Niblett D. (2024). Leveraging Machine Learning in Porous Media. J. Mater. Chem. A.

[105] Zhou L., Sun H., Fan D., Zhang L., Imani G., Fu S., Yang Y., Zhang K., Yao J. (2024). Flow Prediction of Heterogeneous Nanoporous Media Based on Physical Information Neural Network. Gas Sci. Eng..

[106] Alhadri M., Raza J., Yashkun U., Lund L.A., Maatki C., Khan S.U., Kolsi L. (2022). Response Surface Methodology (RSM) and Artificial Neural Network (ANN) Simulations for Thermal Flow Hybrid Nanofluid Flow with Darcy-Forchheimer Effects. J. Indian Chem. Soc..

[107] Raja M.A.Z., Shoaib M., Khan Z., Zuhra S., Saleel C.A., Nisar K.S., Islam S., Khan I. (2022). Supervised Neural Networks Learning Algorithm for Three Dimensional Hybrid Nanofluid Flow with Radiative Heat and Mass Fluxes. Ain Shams Eng. J..

[108] Ishola O., Vilcáez J. (2022). Machine Learning Modeling of Permeability in 3D Heterogeneous Porous Media Using a Novel Stochastic Pore-Scale Simulation Approach. Fuel.

[109] Purcell W.R. (1949). Capillary Pressures—Their Measurement Using Mercury and the Calculation of Permeability Therefrom. J. Pet. Technol..

[110] McNamara G.R., Zanetti G. (1988). Use of the Boltzmann Equation to Simulate Lattice-Gas Automata. Phys. Rev. Lett..

[111] Monaghan J.J. (1992). Smoothed Particle Hydrodynamics. Annu. Rev. Astron. Astrophys..

[112] Øren P.-E., Bakke S., Arntzen O.J. (1998). Extending Predictive Capabilities to Network Models. SPE J..

[113] Piri M., Blunt M.J. (2005). Three-Dimensional Mixed-Wet Random Pore-Scale Network Modeling of Two-and Three-Phase Flow in Porous Media. II. Results. Phys. Rev. E—Stat. Nonlinear Soft Matter Phys..

[114] Piri M., Blunt M.J. (2005). Three-Dimensional Mixed-Wet Random Pore-Scale Network Modeling of Two-and Three-Phase Flow in Porous Media. I. Model Description. Phys. Rev. E—Stat. Nonlinear Soft Matter Phys..

[115] Raeini A.Q., Blunt M.J., Bijeljic B. (2012). Modelling Two-Phase Flow in Porous Media at the Pore Scale Using the Volume-of-Fluid Method. J. Comput. Phys..

[116] Jettestuen E., Helland J.O., Prodanović M. (2013). A Level Set Method for Simulating Capillary-controlled Displacements at the Pore Scale with Nonzero Contact Angles. Water Resour. Res..

[117] Ungerer P., Lachet V., Tavitian B. (2006). Applications of Molecular Simulation in Oil and Gas Production and Processing. Oil Gas Sci. Technol.-Rev. IFP.

[118] Welch W.R., Piri M. (2015). Molecular Dynamics Simulations of Retrograde Condensation in Narrow Oil-Wet Nanopores. J. Phys. Chem. C.

[119] Didar B.R., Akkutlu I.Y. Pore-Size Dependence of Fluid Phase Behavior and Properties in Organic-Rich Shale Reservoirs. Proceedings of the SPE: International Symposium on Oilfield Chemistry.

[120] Frisch U., Hasslacher B., Pomeau Y. (1986). Lattice-Gas Automata for the Navier-Stokes Equation. Phys. Rev. Lett..

[121] Rothman D.H., Zaleski S. (1994). Lattice-Gas Models of Phase Separation: Interfaces, Phase Transitions, and Multiphase Flow. Rev. Mod. Phys..

[122] Zhu J., Ma J. (2018). Extending a Gray Lattice Boltzmann Model for Simulating Fluid Flow in Multi-Scale Porous Media. Sci. Rep..

[123] Guo Z., Zhao T. (2002). Lattice Boltzmann Model for Incompressible Flows through Porous Media. Phys. Rev. E.

[124] Kang Q., Zhang D., Lichtner P.C., Tsimpanogiannis I.N. (2004). Lattice Boltzmann Model for Crystal Growth from Supersaturated Solution. Geophys. Res. Lett..

[125] Asadolahpour S.R., Jiang Z., Lewis H., Chao M. (2024). Deep Learning for Pore-Scale Two-Phase Flow: Modelling Drainage in Realistic Porous Media. Pet. Explor. Dev..

[126] Wang H., Su Y., Wang W., Jin Z., Chen H. (2022). CO_2_-Oil Diffusion, Adsorption and Miscible Flow in Nanoporous Media from Pore-Scale Perspectives. Chem. Eng. J..

[127] Zhang D., Li S., Li Y. (2021). Lattice Boltzmann Simulation of Three Phase Reactive Flow in Random Porous Media at Pore-Scale. Appl. Therm. Eng..

[128] Mahrous M., Curti E., Churakov S.V., Prasianakis N.I. (2022). Petrophysical Initialization of Core-Scale Reactive Transport Simulations on Indiana Limestones: Pore-Scale Characterization, Spatial Autocorrelations, and Representative Elementary Volume Analysis. J. Pet. Sci. Eng..

[129] Jiang M., Xu Z., Zhou Z. (2021). Pore-Scale Investigation on Reactive Flow in Porous Media Considering Dissolution and Precipitation by LBM. J. Pet. Sci. Eng..

[130] Wang H., Cai J., Su Y., Jin Z., Zhang M., Wang W., Li G. (2023). Pore-Scale Study on Shale Oil–CO_2_–Water Miscibility, Competitive Adsorption, and Multiphase Flow Behaviors. Langmuir.

[131] Shan X., Chen H. (1993). Lattice Boltzmann Model for Simulating Flows with Multiple Phases and Components. Phys. Rev. E.

[132] Liu Z., Wu H. (2016). Pore-Scale Modeling of Immiscible Two-Phase Flow in Complex Porous Media. Appl. Therm. Eng..

[133] McDonald P., Turner M. (2015). Combining Effective Media and Multi-Phase Methods of Lattice Boltzmann Modelling for the Characterisation of Liquid-Vapour Dynamics in Multi-Length Scale Heterogeneous Structural Materials. Model. Simul. Mater. Sci. Eng..

[134] Ou Z., Xue Q., Wan Y., Wei H., Liu L., Gharibi F., Thévenin D. (2024). A One-Field Fluid/Meso-Structure Coupling Approach for Multiscale Transport in Heterogeneous Porous Media. Phys. Fluids.

[135] Chen S., Doolen G.D. (1998). Lattice Boltzmann Method for Fluid Flows. Annu. Rev. Fluid Mech..

[136] Saeibehrouzi A., Abolfathi S., Denissenko P., Holtzman R. (2024). Pore-Scale Modeling of Solute Transport in Partially-Saturated Porous Media. Earth-Sci. Rev..

[137] Zhao J., Wu J., Wang H., Xia Y., Cai J. (2024). Single Phase Flow Simulation in Porous Media by Physical-Informed Unet Network Based on Lattice Boltzmann Method. J. Hydrol..

[138] Wang D., Liu F., Sun J., Li Y., Wang Q., Jiao Y., Song K., Wang S., Ma R. (2023). Lattice-Boltzmann Simulation of Two-Phase Flow in Carbonate Porous Media Retrieved from Computed Microtomography. Chem. Eng. Sci..

[139] Choi C.-S., Lee Y.-K., Song J.-J. (2020). Equivalent Pore Channel Model for Fluid Flow in Rock Based on Microscale X-Ray CT Imaging. Materials.

[140] Jin Y. (2024). A Pore-Scale Resolved Direct Numerical Simulation Study for Scaling Analysis of the Solutal Convection in Porous Media. J. Fluid Mech..

[141] Hoang H., Galliero G. (2012). Local Viscosity of a Fluid Confined in a Narrow Pore. Phys. Rev. E—Stat. Nonlinear Soft Matter Phys..

[142] Zhang L., Tan M., Liu X., Lu X., Wang Q., Wang S., Tian M., Wang J. (2025). Insights into Adsorption Behaviors of Multi-Component Shale Oil in Illite Nanopores under Different Reservoir Conditions by Molecular Simulation. Nanomaterials.

[143] Zhao Y., Yang L., Xiao P., Liang Y., Hua X., Tian W., Fang W., Liu B. (2024). Molecular Insight into CO_2_ Improving Oil Mobility in Shale Inorganic Nanopores Containing Water Films. Langmuir.

[144] Xu Z., Wu S., Tian S., Huang D., Xiong G., Luo T. (2023). Molecular-Level Understanding of the Effect of Water on Oil Transport in Graphene Nanochannels. J. Phys. Chem. C.

[145] Yu H., Chen J., Zhu Y., Wang F., Wu H. (2017). Multiscale Transport Mechanism of Shale Gas in Micro/Nano-Pores. Int. J. Heat Mass Transf..

[146] Wu K., Chen Z., Li J., Lei Z., Xu J., Wang K., Li R., Dong X., Peng Y., Yang S. (2019). Nanoconfinement Effect on N-Alkane Flow. J. Phys. Chem. C.

[147] Barisik M., Beskok A. (2014). Scale Effects in Gas Nano Flows. Phys. Fluids.

[148] Hoang H., Galliero G. (2012). Shear Viscosity of Inhomogeneous Fluids. J. Chem. Phys..

[149] Chen G., Liu Z. (2021). Effect of Modification on the Fluid Diffusion Coefficient in Silica Nanochannels. Molecules.

[150] Xiong H., Devegowda D., Huang L. (2020). Water Bridges in Clay Nanopores: Mechanisms of Formation and Impact on Hydrocarbon Transport. Langmuir.

[151] Babu J.S., Sathian S.P. (2011). The Role of Activation Energy and Reduced Viscosity on the Enhancement of Water Flow through Carbon Nanotubes. J. Chem. Phys..

[152] Köhler M.H., da Silva L.B. (2016). Size Effects and the Role of Density on the Viscosity of Water Confined in Carbon Nanotubes. Chem. Phys. Lett..

[153] Alexiadis A., Kassinos S. (2008). Molecular Simulation of Water in Carbon Nanotubes. Chem. Rev..

[154] Zhang H., Ye H., Zheng Y., Zhang Z. (2011). Prediction of the Viscosity of Water Confined in Carbon Nanotubes. Microfluid. Nanofluid..

[155] Ghorbanian J., Beskok A. (2016). Scale Effects in Nano-Channel Liquid Flows. Microfluid. Nanofluid..

[156] Sendner C., Horinek D., Bocquet L., Netz R.R. (2009). Interfacial Water at Hydrophobic and Hydrophilic Surfaces: Slip, Viscosity, and Diffusion. Langmuir.

[157] Bakli C., Chakraborty S. (2015). Slippery to Sticky Transition of Hydrophobic Nanochannels. Nano Lett..

[158] Wu K., Chen Z., Xu J., Hu Y., Li J., Dong X., Liu Y., Chen M. (2016). A Universal Model of Water Flow Through Nanopores in Unconventional Reservoirs: Relationships Between Slip, Wettability and Viscosity.

[159] Kobayashi Y., Arai N., Yasuoka K. (2022). Correlation between Ordering and Shear Thinning in Confined OMCTS Liquids. J. Chem. Phys..

[160] Liu H., Xiong H., Yu H., Wu K. (2022). Effect of Water Behaviour on the Oil Transport in Illite Nanopores: Insights from a Molecular Dynamics Study. J. Mol. Liq..

[161] Xie Q., Alibakhshi M.A., Jiao S., Xu Z., Hempel M., Kong J., Park H.G., Duan C. (2018). Fast Water Transport in Graphene Nanofluidic Channels. Nat. Nanotechnol..

[162] Li Y., Xu J., Li D. (2010). Molecular Dynamics Simulation of Nanoscale Liquid Flows. Microfluid. Nanofluid..

[163] Nazari M., Davoodabadi A., Huang D., Luo T., Ghasemi H. (2020). Transport Phenomena in Nano/Molecular Confinements. ACS Nano.

[164] Abdelrazik A., Sayed M.A., Omar A.M., FM F.A., Alshimaa H., Oulguidoum A., Kotob E., Helmy M.H. (2023). Potential of Molecular Dynamics in the Simulation of Nanofluids Properties and Stability. J. Mol. Liq..

